# Biogenic Zinc Oxide Nanoparticles synthesized from *Tinospora Cordifolia* induce oxidative stress, mitochondrial damage and apoptosis in Colorectal Cancer

**DOI:** 10.7150/ntno.84995

**Published:** 2024-03-09

**Authors:** Hadgu Mendefro Berehu, Srinivas Patnaik

**Affiliations:** Disease Biology Laboratory, School of Biotechnology KIIT Deemed to Be University, Odisha, India.

**Keywords:** Anticancer, Apoptosis, biogenic ZnO-NPs, Caspase cascade, Colorectal cancer, Cytotoxicity, Green synthesis, *in vivo*, MMP, ROS

## Abstract

Cancer chemotherapy remains a serious challenge, and new approaches to therapy are urgently needed to build novel treatment regimens. The methanol extract of the stem of *Tinospora Cordifolia* was used to synthesize biogenic zinc oxide nanoparticles (ZnO-NPs) that display anticancer activities against colorectal cancer. Biogenic ZnO-NPs synthesized from methanol extract of *Tinospora Cordifolia* stem (ZnO-NPs TM) were tested against HCT-116 cell lines to assess anticancer activity. UV-Vis, FTIR, XRD, SEM, and TEM analysis characterized the biogenic ZnO-NPs. To see how well biogenic ZnO-NPs fight cancer, cytotoxicity, AO/EtBr staining, Annexin V/PI staining, mitochondrial membrane potential (MMP), generation of reactive oxygen species (ROS) analysis, and caspase cascade activity analysis were performed to assess the anticancer efficacy of biogenic ZnO-NPs. The IC50 values of biogenic ZnO-NPs treated cells (HCT-116 and Caco-2) were 31.419 ± 0.682μg/ml and 36.675 ± 0.916μg/ml, respectively. qRT-PCR analysis showed that cells treated with biogenic ZnO-NPs Bax and P53 mRNA levels increased significantly (*p* ≤ 0.001). It showed to have impaired MMP and increased ROS generation. In a corollary, our *in vivo* study showed that biogenic ZnO-NPs have an anti-tumour effect. Biogenic ZnO-NPs TM showed both *in vitro* and *in vivo* anticancer effects that could be employed as anticancer drugs.

## 1. Introduction

Colorectal cancer (CRC) is the third most common cause of new cancer cases (1.93 million cases) in 2020, trailing only breast and lung cancer. It was the second leading cause of death (916,000 deaths) in 2020, next to lung cancer 1. Colorectal cancer is a complex and aggressive disease that poses significant challenges for researchers and healthcare professionals. The incidence of colorectal cancer is on the rise, particularly among young adults, highlighting the urgent need for effective treatment options and prevention initiatives 2. While conventional cancer therapies, such as chemotherapy and radiation, have been crucial in managing the disease, they often come with significant side effects and limited efficacy 3, 4. Although several anticancer chemotherapies are currently available, they fail to produce a complete anticancer response due to drug resistance or their inability to differentiate between healthy and cancerous cells accurately, and they often require large amounts of drug administration 5. Traditional chemotherapy kills rapidly proliferating cancer cells by interfering with their ability to synthesize DNA. The chemicals are not discriminating, so they could cause harm to normal tissues as well as cancerous ones, leading to serious unwanted side effects, including nausea and weight loss.

In recent years, nanoparticles have emerged as a promising avenue for cancer treatment, offering targeted delivery of therapeutic agents and enhanced treatment efficacy. Numerous studies have explored the use of various types of nanoparticles, such as gold nanoparticles, Carbon-based NPs, Lipid-based NPs, Polymeric NPs, cobalt oxide nanoparticles, Ag NPs, CuO NPs, ZnO NPs, MgO NPs, CaO NP, Fe NPs etc, in the treatment of cancer 6, 7, 8, 9, 10. These nanoparticles have shown potential in improving the bioavailability of polyphenols, protecting and delivering medicine to the colon, and integrating controlled release mechanisms for enhanced chemotherapy efficacy 11. Their unique properties, such as small size and large surface area, allow for efficient and precise delivery of drugs to cancer cells while minimizing damage to healthy tissues 12. Furthermore, in order to boost safety and efficacy, NPs may enhance the stability and solubility of encapsulated cargos, facilitate transport across membranes, and extend circulation times 13, 14.

Because of their biocompatibility, zinc oxide nanoparticles (ZnONPs) are one of the metal oxide nanoparticles that are gaining popularity as a semiconductor nanomaterial with a wide variety of applications in photocatalysis, cosmetics, electrical and optoelectronic devices, and most crucially, biomedicine. They do have large band-gap, high exciton energy, and rapid electron mobility, that allows it to have special physical and chemical properties 15. ZnO-NPs have specific cytotoxicity towards cancer cells, as demonstrated by several *in vitro* studies 16-17. In comparison to normal cells, Hanley proposed that they exhibit 28-35 times more selective toxicity against cancer cells 18. By specifically directing ZnO-NPs toward cancer cells, it is possible to further capitalize on this selective cytotoxicity in cancer cells in an in vitro setting. ZnO-NPs have been shown in several studies to exhibit selective cytotoxicity against cancer cells; nevertheless, the precise mechanism behind this selectivity remains unknown. Thus, the objective of this research paper is to provide an in-depth analysis of the effect of biogenic ZnO-NPS on colorectal cancer and evaluate its potential as a medical intervention.

The green synthesis of ZnO-NPs was discovered to significantly reduce the need for metal ions, expensive chemicals, and time-consuming experimental techniques. *Tinospora Cordifolia* (*T. Cordifolia*) is a medicinal plant that is a genetically varied, large, deciduous climbing shrub with greenish-yellow flowers that grow at higher altitudes. This plant is common in India and has various therapeutic benefits traditionally 19. However, a review of research findings showed that the *T. Cordifolia* plant extract has never been employed to synthesize ZnO-NPs.

Over the last two decades, substantial research efforts have been made toward developing eco-friendly and cost-effective nanomaterials 20. Herbs are the most widely utilized sources for NP synthesis because of their availability, accessibility, and rich source of reducing agents 20, 21. In this article, we hypothesize that eco-friendly and cost-effective biogenic ZnO-NPs can be synthesized from the local medicinal plant *T. Cordifolia* extract and this synthesized biogenic ZnO-NPs can kill colorectal cancer cells via ROS-induced mitochondrial membrane potential disruption mechanism. This study was evaluated by conducting both *in vitro* and *in vivo* experiments. We conducted cytotoxicity, apoptosis induction, ROS imbalance, mitochondrial potential disruption as well as the tumor reduction, both in volume and weight in mice, in our *in vivo* study. we seek to provide valuable insights into the promising role of biogenic ZnO-NPs as an alternative therapy in the management of colorectal cancer.

## 2. Materials and Methods

### 2.1. Chemicals and instruments

The Zinc nitrate hexahydrate [Zn (NO_3_)_2·_6H_2_O] was purchased from Sigma-Aldrich, (CAS #: 10196-18-6, Sigma-Aldrich, USA); whereas methanol (CAS #: 67-56-1) and Acridine orange (CAS #: 10,127-02-3) were purchased from SRL; Cellular ROS assay kit (red) (CAS #: ab186027, Abcam, UK) and JC-1-mitochondrial membrane potential assay kit (CAS #: ab113850, Abcam, UK) purchased from Abcam; Annexin V-FITC apoptosis detection kit (Cat #: 1001K) was purchased from Abgenex for apoptotic and necrotic studies ( Abgenex Plc, Bhubaneswar, India ); and sodium hydroxide (CAS #: 1310-73-2) were obtained from Merck. Cellular ROS Assay Kit (Red) (ab186027) and JC-1 - Mitochondrial Membrane Potential Assay Kit (ab113850) were purchased from Abcam (Abcam plc, Cambridge, United Kingdom). To analyse qualitative and quantitative the assessments the following technologies are used: Gas chromatography-mass spectrometer (GC-MS) (Agilent 7890A, United States), UV-visible spectroscopy (Cary 100 UV-Vis Spectrophotometer, United States), FTIR (100 Spectrum, Perkin Elmer, Germany), XRD (Rigaku Smart Lab 8A Series, Japan), high-resolution TEM (JEM-2100, HRTEM, JEOL, JAPAN), bench top fluorescence microscopy (FLoid™ Cell Imaging Station, United States), BD FACS Canto II flow cytometer (BD Biosciences, United States), and centrifuge (5804/5804R Eppendorf benchtop centrifuge, United States).

### 2.2. Cell Culture

The National Center for Cell Science (NCCS) in Pune, India, supplied the cell lines (colorectal cancer cell lines Caco-2 and HCT-116 as well as control cells HEK-293). In DMEM, which contains 10% heat-inactivated FBS, 1% 1-glutamine, and 1% antibiotics (Penicillin/Streptomycin), the cells were cultured for 24 hours in a humidified 37°C (5% CO_2_) incubator.

### 2.3. Preparation of plant extract

Fresh stems of *Tinospora cordifolia* were collected from the Balasore district of Odisha State, India. The stem was thoroughly washed with tap water and deionized water three times before being chopped and dried in the shade away from direct sunlight for seven days. The dried chopped stems were ground to a powder using an electric grinder. As per previous extraction techniques, one gram of the finely powdered stem of *T. cordifolia* was immersed in 100 ml of 90% methanol and placed in a hot water bath for 3-4 h 21. After filtering through a 0.45-μm Whatman filter paper, the extract was concentrated in a rotary flash evaporator at temperatures between 35 and 40 °C. Once the extract was concentrated, it was placed in airtight bottles and kept at 4°C until required.

### 2.4 Analysis using Gas Chromatography-Mass Spectrometry (GC-MS)

*Tinospora cordifolia* has been reported to have a wide range of compounds, including polysaccharides, alkaloids, cadinene sesquiterpenes, and phenylpropanoid glycosides 19. The phytoconstituents of a methanol extract of *Tinospora cordifolia* stems were looked at with an Agilent 7890A gas chromatograph connected to a mass spectrometer (GC-MS) with an autosampler and a fused silica column with an Elite-5MS capillary column (30 m x 0.25 mm I.D. x 25 m). The carrier gas was 99.99 % pure helium gas, flowing at a steady rate of 1 ml/min with an average velocity of 36.445 cm/s throughout the experiment's 32-minute duration 22. For GC-MS spectrum detection, an electron mode at 70 eV and a scan time of 0.2 seconds with fragments ranging from 40 to 600 m/z were used. One liter of mobile phase was injected at a temperature of 250 degrees Celsius (a split ratio of 10:1). After being held at 50 degrees Celsius for 1.3719 minutes, the temperature in the column oven was increased by 10 degrees every minute until it reached 250°C. Next, the temperature was increased to 325°C for 32 minutes. The phytoconstituents in the plant extract were compared with the spectra database of authenticating compounds held by the National Institute of Standards and Technology (NIST) 08 Mass Spectral Library, looking for retention time, peak height, peak area, and mass spectra patterns.

### 2.5. Synthesis of biogenic Zinc Oxide Nanoparticles (ZnO-NPs)

Biogenic ZnO-NPs were synthesized as described in earlier works 23. In a 250-ml flask, 100 ml of 1 mg/ml plant extract was dissolved in 100 ml of zinc nitrate hexahydrate. 100 ml of 200 mM NaOH aqueous solution was driped into the mixture with a burette and constantly stirred for 3 hours on a magnetic stirrer (70°C). The mixture was then left to settle down overnight. After discarding the supernatant, the light yellow-colored pellet was kept. The pellet was centrifuged at 10,000 rpm for ten minutes after being flushed with deionized water three times. The precipitates generated were then dried in a hot air oven at 100 °C overnight to obtain a creamy paste of ZnO-NPs. Dry ZnO-NPs were powdered using a mortar and pestle.

### 2.6. Characterization of biogenic ZnO-NPs

A UV-visible absorption spectrophotometer, FT-IR, XRD, SEM, and TEM were used to characterize the biogenic ZnO-NPs synthesized from methanolic extract of *Tinospora cordifolia* stem.

#### 2.6.1. UV-Visible Analysis

To assess the presence of synthesized ZnO-NPs, their optical absorbance was analyzed using a Cary 100 UV-vis spectrophotometer.

#### 2.6.2. Fourier transform infrared spectroscopy (FT-IR)

FT-IR was used to investigate the interaction of functional groups of plant extract compounds conjugated with ZnO nanoparticles. The lyophilized nanoparticle sample was combined with a potassium bromide pellet and exposed to pressure as per the standards, and the range was established at 1 cm^-1^ progress 24. IR spectra in the frequency range of 4000-400 cm^-1^ were acquired at RT.

#### 2.6.3. X-Ray Diffraction (XRD) Analysis

The crystalline structure of dried powder of biogenic ZnO nanoparticles was characterized by XRD. The spectra were determined to be 40 kV at 30 mA. The diffraction pattern was registered over a 2θ range of 5°-90°. The Debye-Scherrer technique was used to compute the average size of the green synthesized ZnO-NPs (Dp = 0.9λ/βCosθ), where D is the average crystallite size, β denotes the line broadening the value of FWHM of a peak, λ is the wavelength of the irradiation X-rays (λ = 0.15406 nm), K is the shape factor typically taken as 0.9, and θ is the maximum peak position value. The obtained diffractogram was similar to (JPCDS card number: 36-1451) the XRD reference data for ZnO-NPs of the International Center for Diffraction Data (ICDD) 25.

#### 2.6.4. Scanning Electron Microscopy (SEM)

The morphology of biogenic ZnO-NPs was assessed using SEM (Carl ZEISS Supra 55, Germany).

#### 2.6.5. Transmission Electron Microscopy (TEM)

TEM was used to view the morphology of green synthesized ZnO-NPs. The biogenic ZnO-NPs were analyzed using HRTEM (HRTEM—JEOL JEM-2100 PLUS, Japan). The ZnO-NPs were diluted in sterile deionized water, and then the suspension was subjected to a carbon-coated copper grid and allowed to dry 26.

### 2.7. Cell cytotoxicity (MTT assay)

The cytotoxicity of biogenic ZnO-NPs on the colorectal cancer cell lines (Caco-2 and HCT-116 cell lines) was assessed using MTT assay. In a 96-well plate, cells were seeded (2 × 10^4^ cells/well in 100 μl of DMEM) and then kept at 37ºC in a humidified environment (5% CO_2_) for 24 hours. After 24 hours of incubation, the cells were treated with different concentrations (5, 25, 50, 100, and 200 μg/ml) of plant extract and their respective synthesized ZnO-NPs. After incubation for 24 hours after treatment, a solution of 0.5 μg/ml MTT was spiked to each well with the cells, the plate was covered with aluminum foil, and the cells were kept at 37ºC 5% CO_2_ for 4 hrs. The medium was taken out, and the purple MTT formazan crystals were dissolved with a solubilization solution following the metabolically active cells converting the substrate into a chromogenic product. Before absorbance was evaluated using a microplate reader, the plate was kept on a rocker for 15 minutes. The measurement was taken at 570 nm wavelength 27. The proportion of cell viability to absorbance was reported as a relative decrease in comparison to the absorbance from the control, which was thought to contain 100% of viable cells.

### 2.8. Acridine Orange (AO)/Ethidium Bromide (EtBr) staining

Acridine orange (AO) stain conjugates with all cells and emits green fluorescence. Ethidium Bromide (EtBr) staining will interact with cells lacking cytoplasmic membranes; the nucleus will look red when interpolating with DNA. Live cells have green nuclei, while cells undergoing early apoptosis have intense green fluorescence observed via benchtop fluorescence microscopy. Condensed chromatin is observed as green patches, while late apoptotic cells are regarded as red-to-orange-coloured cells. Based on previous experience and with some modification of the work done previously 24, 25, the morphology of apoptotic cells was examined using AO/EtBr double staining. The HCT-116 cell lines were seeded in a 6-well plate 24 hours before treatment with crude methanol extract of *T*. *cordifolia* and biogenic ZnO-NPs. After incubation for 24 hours after the treatment, the wells with the cells were washed with 500 μl of PBS. Further, a double staining solution of 50μl containing 100g/ml AO and 100g/ml of EtBr was added to the wells with cells, and then, after 5-10 minutes, the cells were again washed with 500 μl of PBS. The morphology of apoptotic cells was observed using benchtop fluorescence microscopy (FLoid™ Cell Imaging).

### 2.9. RT-qPCR analysis

Real-time qPCR was used to analyze the apoptotic gene markers Bcl-2 and Bax genes. Trizol reagent (Invitrogen) was used to extract total RNA from 6-wells of plates which were treated with crude methanol extract of *T. Cordifolia* and biogenic ZnO-NPs 24 hours prior. Gel electrophoresis was done to affirm the presence and integrity of the RNA. Furthermore, NanoDrop analysis was done to determine the isolated RNA's concentration and purity. cDNA was synthesized according to the instructions of the manufacturer using a Verso cDNA synthesis kit by running it in PCR (Thermo Fisher Scientific). Then the gene of interest was amplified using the synthesized cDNA, specific primers, and as directed by the manufacturer using DyNAmo ColorFlash SYBR Green qPCR Kit (Thermo Fisher Scientific). The GAPDH housekeeping gene was used as a normalizer 23. Relative expressions were determined by applying the 2^-ΔΔCT^ method. The data were presented as the mean ± SEM.

### 2.10. Assessment of reactive oxygen species (ROS)

HCT-116 colorectal cancer cell lines were seeded in a 96-well plate to measure the cellular ROS levels. After cells were treated with crude methanol extract of *T*.* cordifolia* and biogenic ZnO-NPs, they were kept in an incubator for 24 hours. After treatment with biogenic ZnO-NPs, cells were stained with 100µL ROS red working solution per well according to the manufacturer's instruction (Abcam, 186027) and kept for 60 minutes at 37°C. Finally, cells detected the fluorescence signal using a microplate reader equipped with an Ex/Em= 520 nm/605 nm filter and a microplate spectrophotometer 28.

### 2.11. Assessment of Mitochondrial Membrane Potential (MMP)

The JC-1 MMP assay (Abcam, 113850) was used to detect mitochondrial membrane potential. When the cell's membrane stays intact, the dye will be retained inside the cells and give high fluorescence. In contrast, when the membrane potential is altered, there will be reduced fluorescence that indicates compromised mitochondrial membrane integrity 29. JC-1 is added to the cells at a predetermined concentration and incubation time. The dye accumulates in the mitochondria. The red aggregate form of JC-1 is an indicator of healthy, polarized mitochondria, while the green monomeric form represents depolarized mitochondria. To assess the impairment of mitochondrial membrane potential (MMP), cells were seeded 24 hours before treatment in a 6-well plate and incubated with biogenic ZnO-NPs, the corresponding crude methanol extract plant, and an untreated control for 24 hours. Cells were re-suspended in fresh media containing 5 g/mL JC-1 (Abcam, 113850) for 30 minutes at 37°C. Cells were then centrifuged at 800 rpm for 5 minutes before re-suspending in 1m of 1x PBS and evaluated with FACS analysis 30.

### 2.12. Annexin V/PI assay analysis using flow cytometric

The Annexin V/ Propidium iodide (PI) assay was used to assess cells that have undergone apoptosis. The annexin V protein linked to FITC recognized phosphatidylserine externalization as a marker of early-stage apoptosis. In contrast, the binding of PI to nuclear DNA revealed membrane damage. Cells were prepared in 6-well plates (4 x 10^5^ cells/well); 24 hours post-treatment, the cells were extracted and washed with PBS. Per the manufacturer's instruction, the pellet was resuspended with 100 µl 1X Binding Buffer. Then, cells were stained with Annexin V (5µl) and PI (5µl) and kept for 20 minutes at RT in the dark. A further 1X Binding Buffer volume of 400 µl was spiked to each tube. Analysis was done using a BD FACS Canto II flow cytometer (BD Biosciences, USA).

### 2.13. *In vivo* anti-cancer studies

The BALB/c mice that were 5-7 weeks old were obtained from the animal house and research facility of KSBT, Bhubaneswar, India. CT-26 cell lines (1.5 x 10^6^ cells suspended in 1ml solution of matrigel and 1x PBS in 1:1 ratio per mouse) were subcutaneously injected into the right flank of mice to induce colorectal cancer tumor 31. After a palpable solid tumor was revealed (volume around 100 mm^3^), mice were randomly grouped into four groups (each n = 5). The groups were, control set (group one), treated with PBS only (group two), treated with methanol extract of *T. cordifolia* stem (group three), and treated with biogenic ZnO-NPs synthesized from *T. cordifolia* methanol extract stem (group four). Treatment was given subcutaneously near the tumor area every other day. Tumor volume was measured and calculated using the formula [V= (W x L)/2], where V is volume, L is the longest diameter, and W is the shortest diameter. After 10 days of treatment, mice were sacrificed to elucidate the post-mortem examination.

### 2.14. Statistical Analysis

A one-way analysis of variance (ANOVA) was used for statistical data analysis. A mean and standard deviation were used to express the values. Origin, ImageJ, and GraphPad prism software are also used to analyze data. Each experiment was repeated thrice.

## 3. Results

### 3.1. GC-MS Analysis

**Figure 2:**
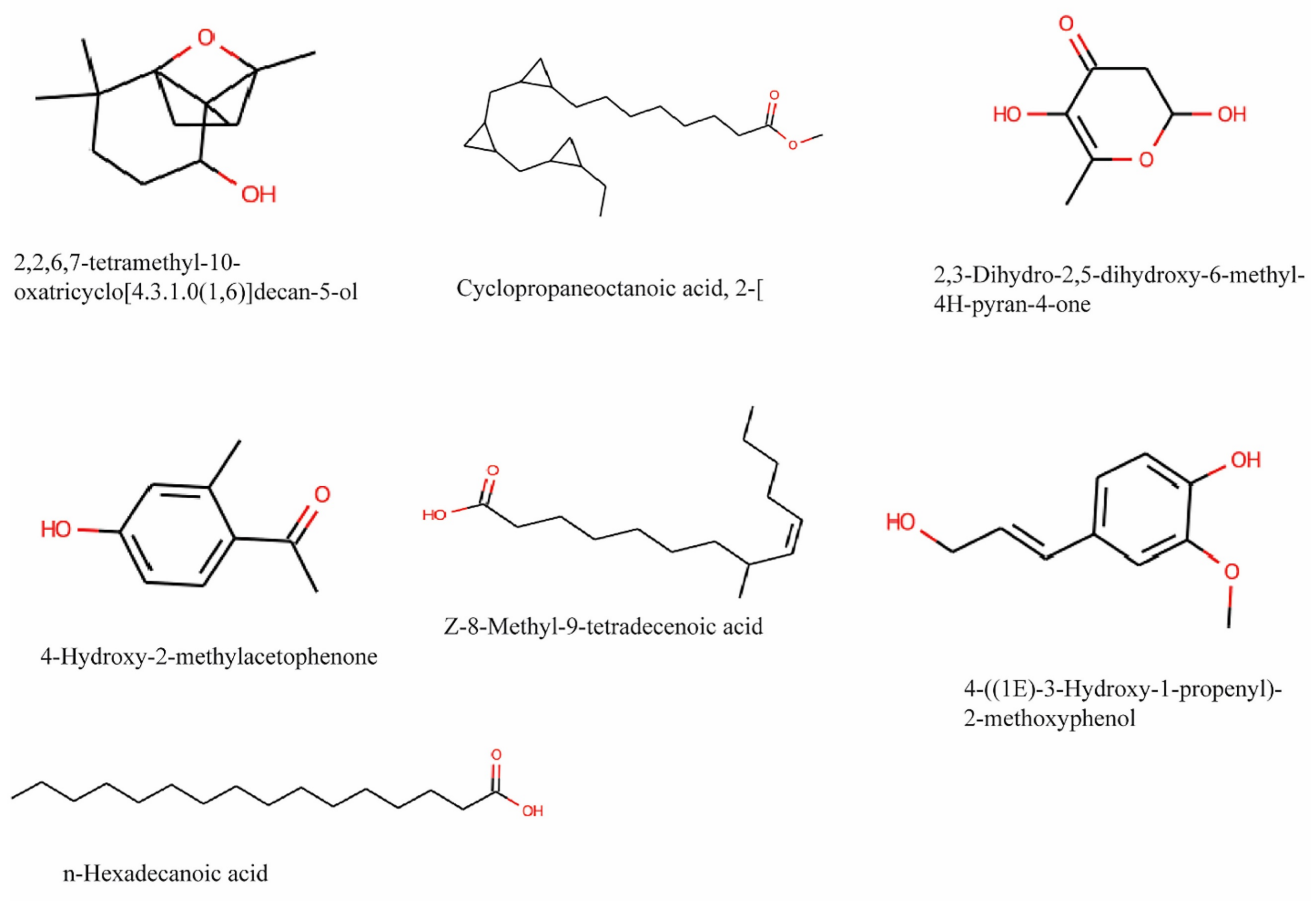
Chemical structure of the main phytocompounds in the methanol extract of stem of *Tinospora cordifolia* identified by GC-MS analysis.

According to different retention times, peak areas, peak heights, and mass spectral patterns in the chromatogram, GC-MS analysis of a 90% methanol extract of *T. cordifolia* stem revealed the presence of 29 compounds (Figure S1 and Table S1). 4-((1E)-3-Hydroxy-1-propenyl)- 2-methoxy, n-Hexadecanoic acid, 8-Pentadecanone, 4-Hydroxy-2-methyl acetophenone, 1-Eicosanol, Cyclo propaneoctanoic acid, Z-8-Methyl-9-tetradecenoic acid, 2,3-dihydro-3,5- dihydroxy, 4H-Pyran-4-one, and 2-[[2-[(2-eth, 2,2,6,7-Tetramethyl-10-oxatricyclo[4.3.1, are the prominent phytoconstituents present in the extraction and their structure is shown in Figure 2. The result was compared to those in the spectra database of authenticating compounds maintained by the National Institute of Standards and Technology (NIST) 08 Mass Spectra Library.

### 3.2. UV-Visible Spectrophotometer Analysis

**Figure 3:**
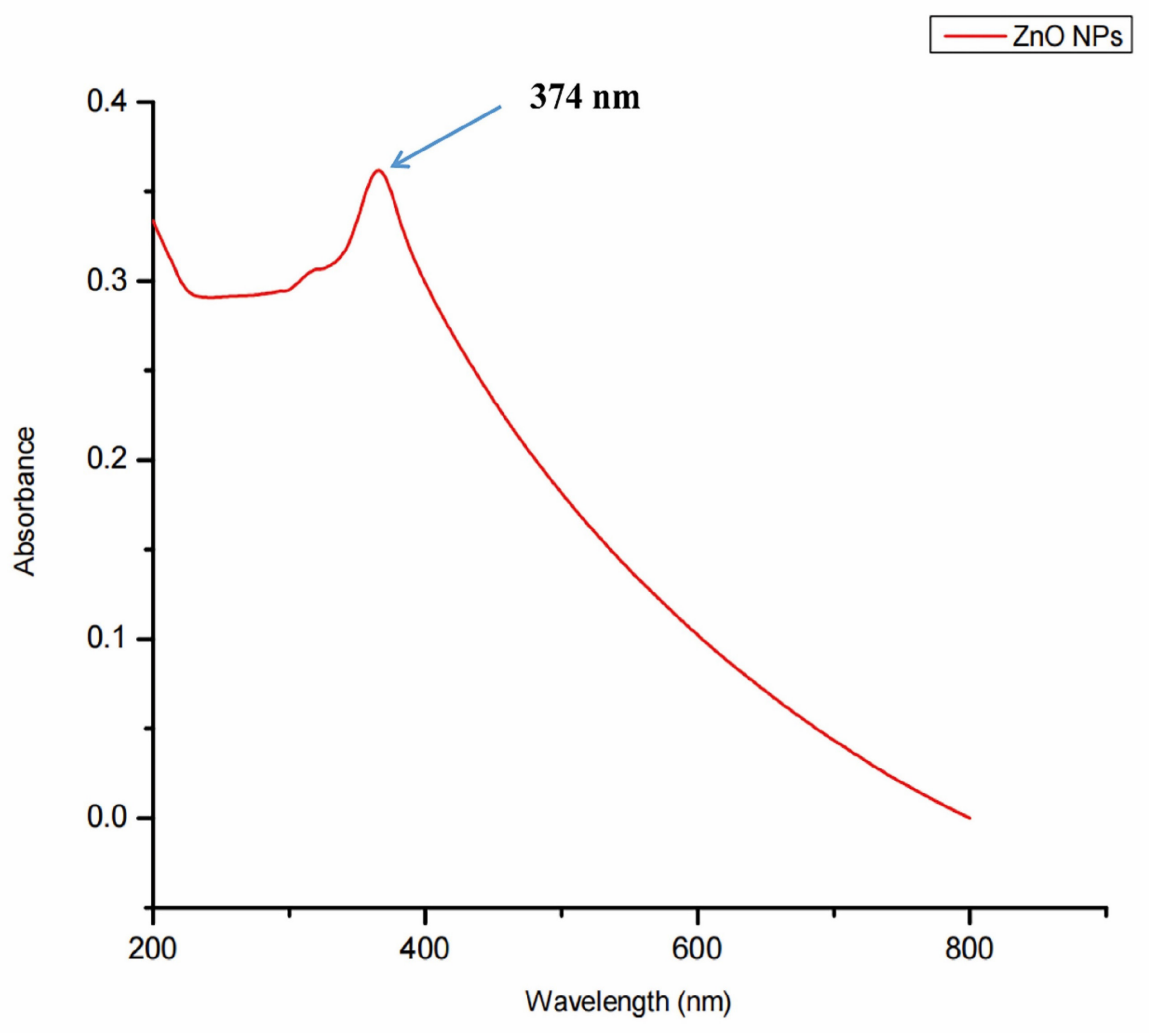
UV-Visible spectra showing the biogenic ZnO-NPs peak located at 374 nm.

Surface plasmon resonance, a collection of excitations on the surface of nanoparticles, was utilized to assure the synthesis, size, and shape of NPs. The biogenic ZnO-NPs diluted in water were identified using a UV-Vis spectrophotometer. At 374 nm, the typical absorption peak of biogenic ZnO-NPs was observed, as shown in Figure 3. The absorption spectrum of ZnO-NPs runs from 200 to 800 nm. The band gap value of the biogenic ZnO-NPs derived from the synthesis data was 3.12 eV.

### 3.3. FTIR Analysis

**Figure 4:**
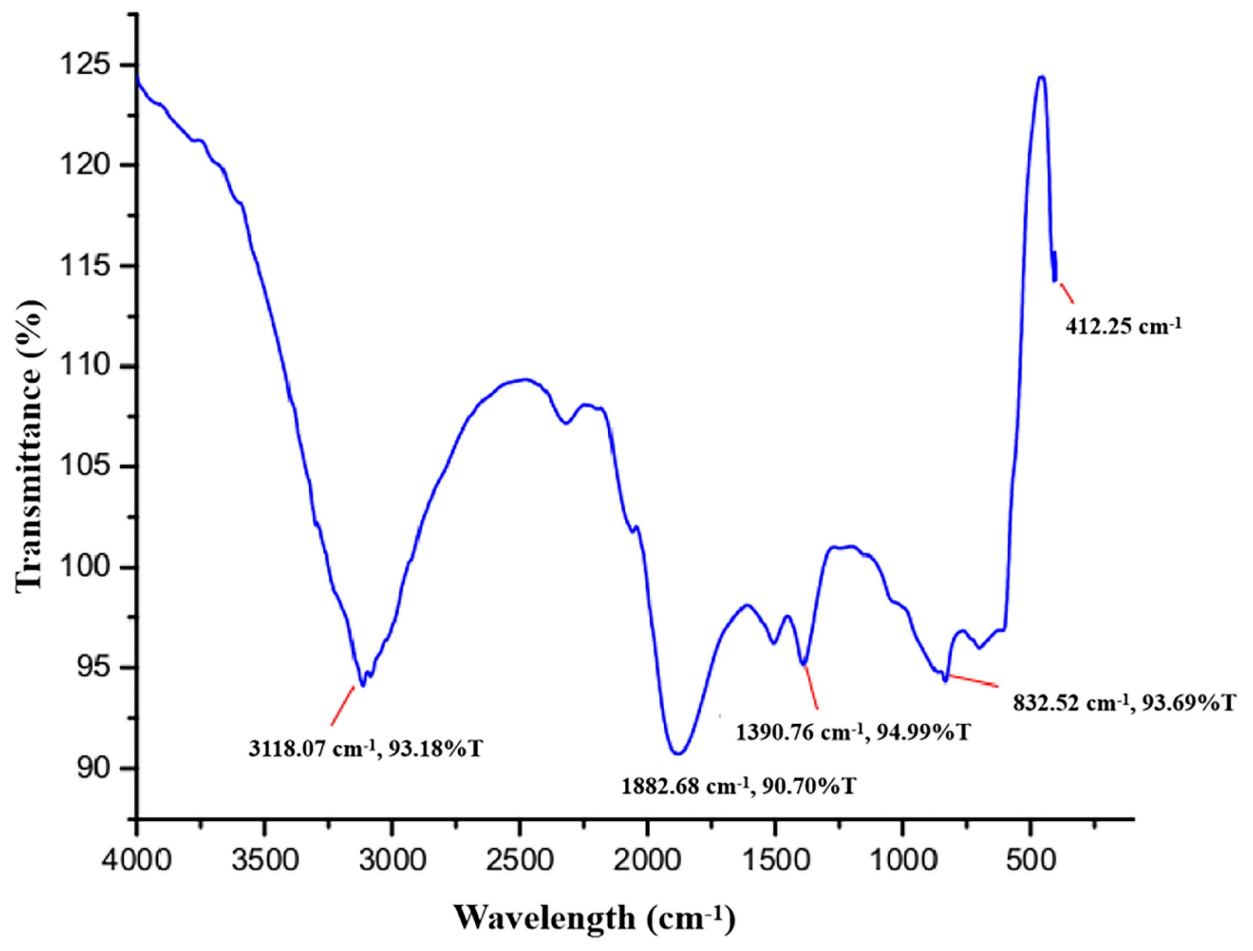
Fourier transform infrared (FTIR) spectra of biogenic ZnO-NPs.

As shown in Figure 4, the principal peaks in the FTIR spectra are arrayed in the wave number region of 4000 to 400 cm^-1^. The bands for functional groups in the spectra of biogenic ZnO-NPs were found at 3118.07 cm^-1^, 1882.68 cm^-1^, 1390.76 cm^-1^, 832.52 cm^-1^, and 412.25 cm^-1^. The vibration of C-H stretching groups causes the wide range peak in the upper area at 3118.07 cm^-1^, which corresponds to the alkene compound classes. The carbonyl compound with the stretching of the C-H bending group peaks at 1882.68 cm^-1^. At 1390.76 cm^-1^, the C-H bending vibration appears that corresponds to aldehyde compounds on the surface of the the NPs.

### 3.4. XRD Analysis

**Figure 5:**
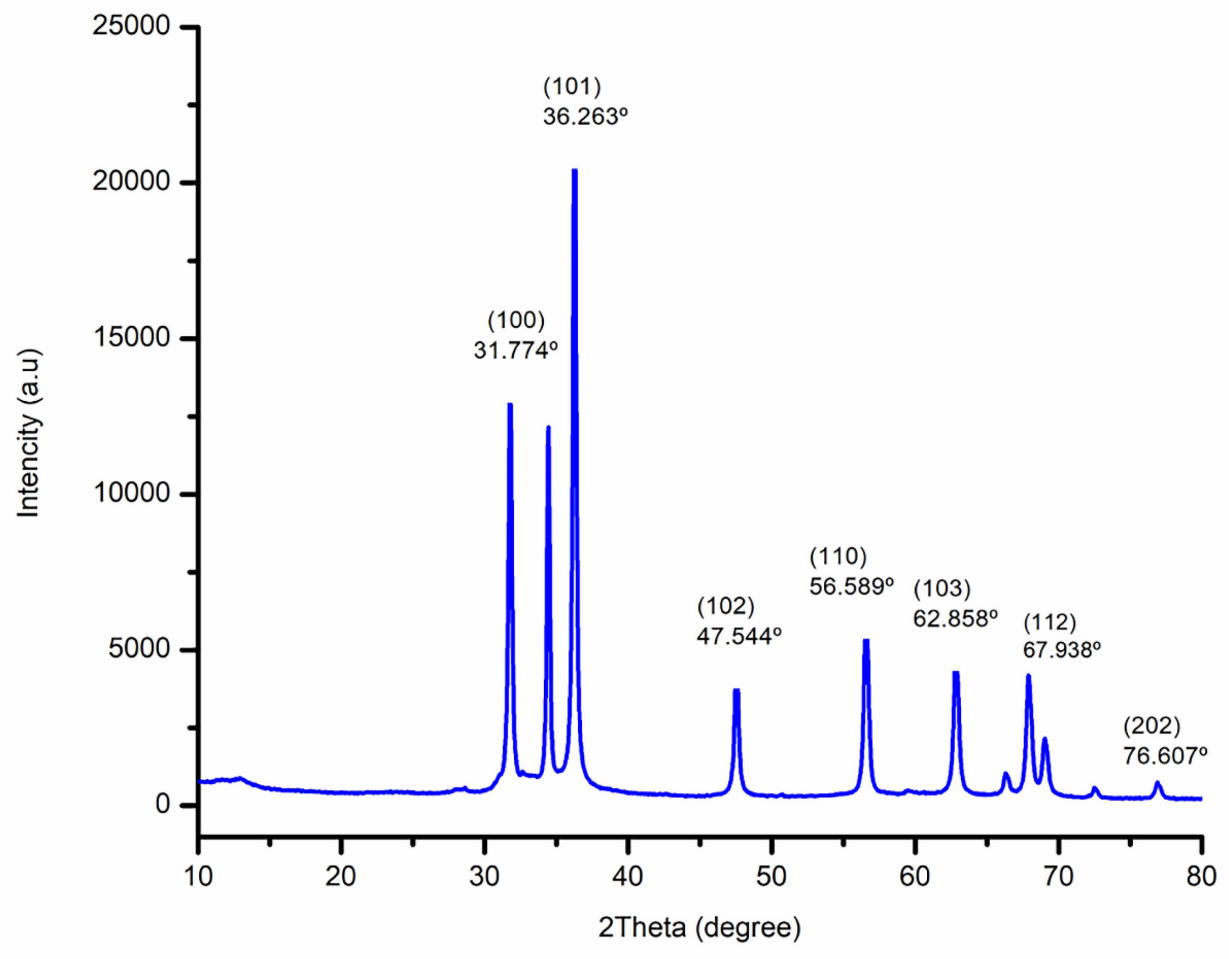
X-Ray Diffraction (XRD) spectra of biogenic ZnO-NPs.

The XRD patterns of biogenic ZnO-NPs from the methanol extract of *T*.* cordifolia* stem were ascribed to hexagonal Wurtzite. As shown in (Figure 5), the analysis revealed that the peaks (2θ) at 31.774º, 34.440º, 36.263º, 47.544º, 56.589º, 62.858º, 66.362º, 67.938º, 69.058º, 72.66º, and 76.607º for (100), (002), (101), (102), (110), (103), (200), (112), (201), (004), and (202) planes of the crystal lattice, respectively. Bragg's equation computed values of the d-spacing values for the main peaks and the lattice parameters for the hexagonal crystalline structure were (a = b = 0.328 nm and c = 0.522 nm). The average crystallite size of biogenic ZnO-NPs was 46.431 nm.

### 3.5. Scanning Electron Microscopy (SEM) Analysis

**Figure 6:**
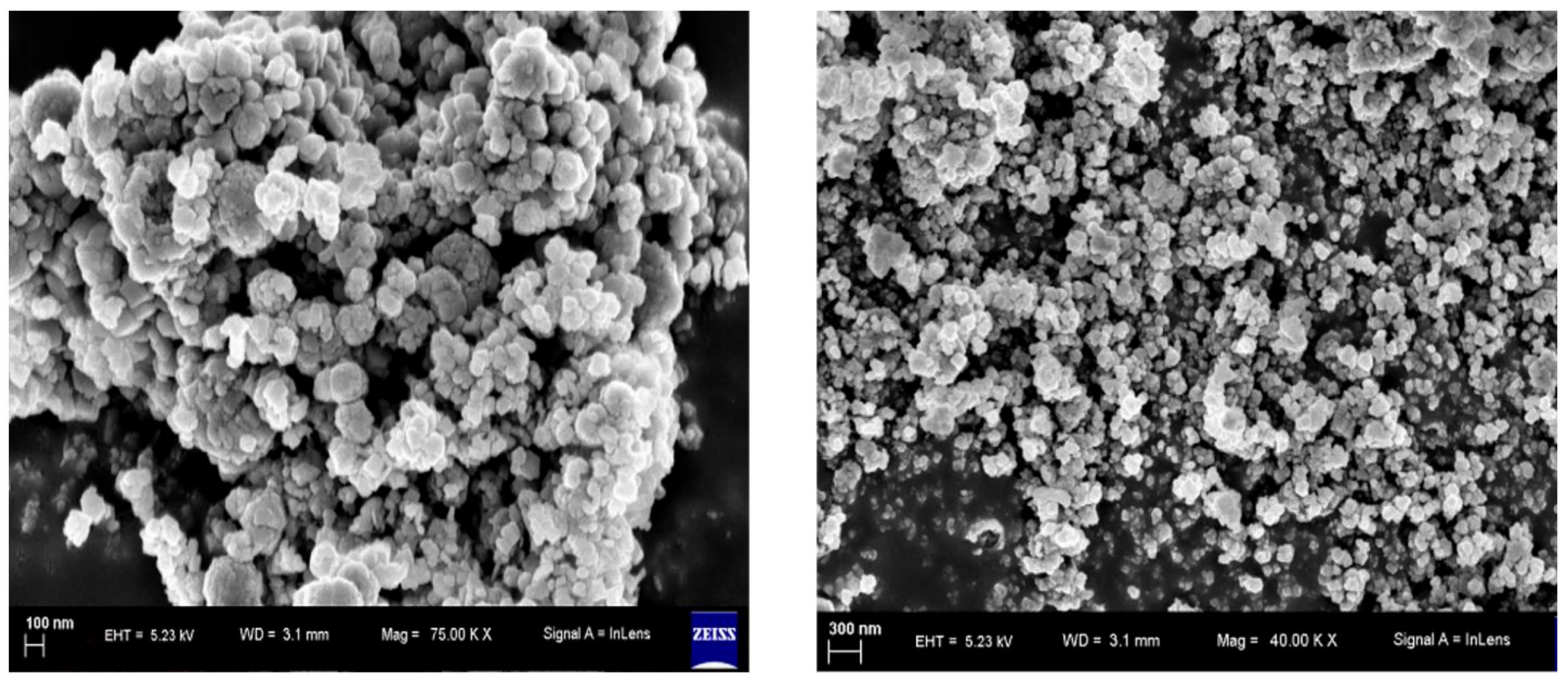
SEM spectra of biogenic ZnO nanoparticles.

SEM analysis was conducted to examine the surface morphology and particle size of the biogenic ZnO-NPs. As shown in Figure 6, the particles have a sphere-shaped morphology with a high degree of aggregation and a spongy-like appearance topographically.

### 3.6. Transmission Electron Microscopy (TEM) Analysis

**Figure 7:**
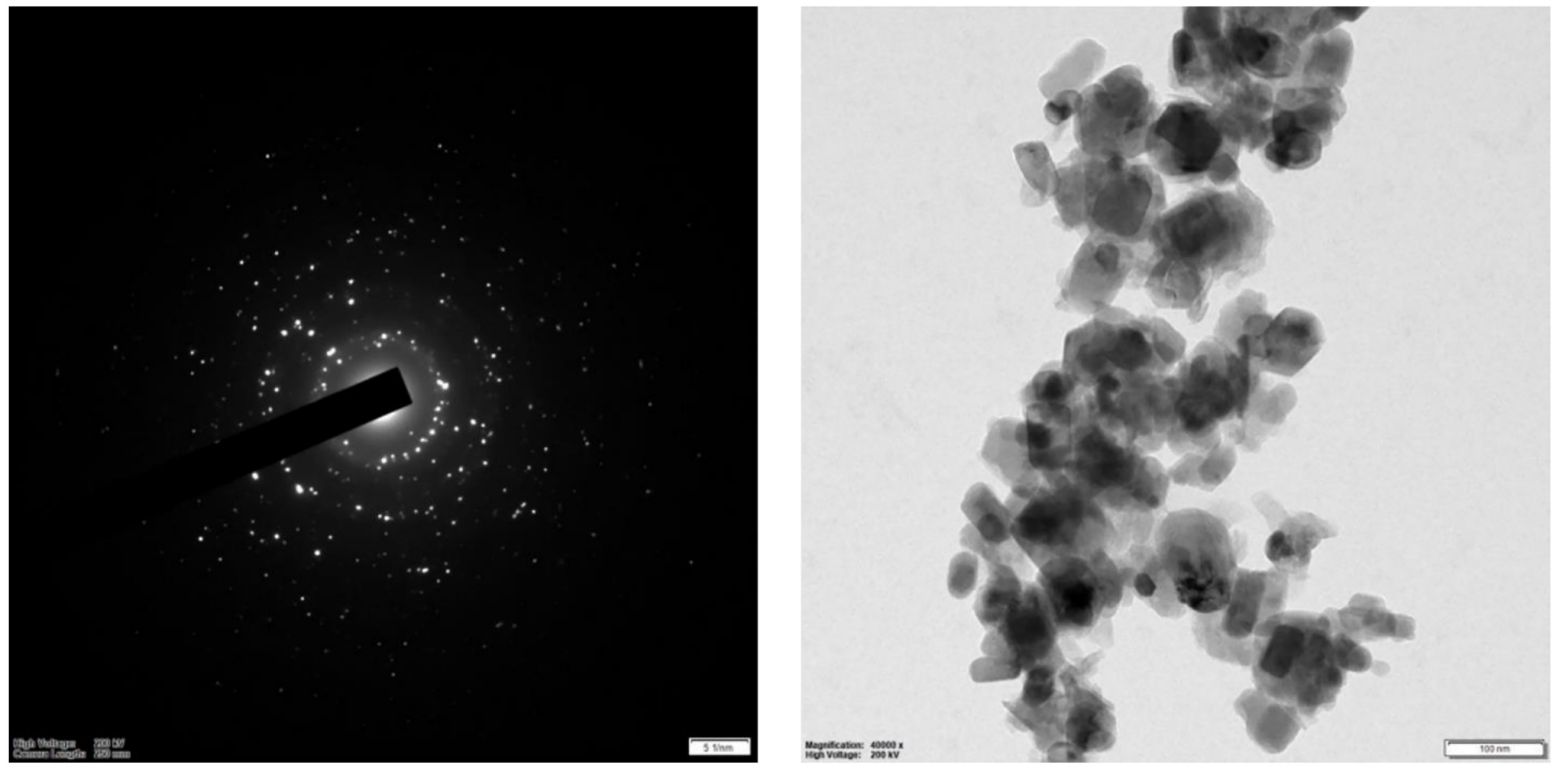
HR-TEM spectra of biogenic ZnO nanoparticles.

The structure and size distribution of biogenic ZnO-NPs were analyzed using TEM, as shown in Figure 7. The analysis showed that the average particle size is 29.5 nm.

### 3.7. Cytotoxicity activity of biogenic ZnO nanoparticles

**Figure 8:**
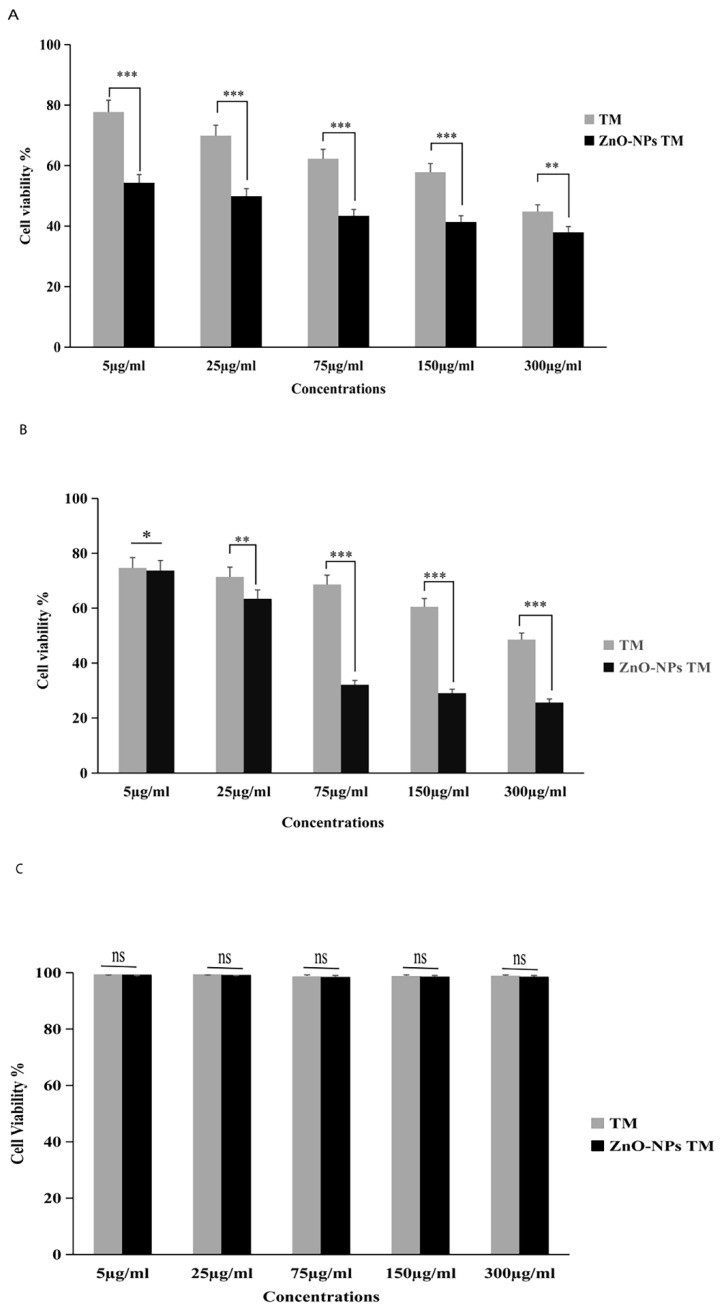
Relative viability percent of colorectal cancer cell lines (HCT-116 and Caco-2) and control cell line HEK-293 after 24 h incubation with various concentrations of methanol extract of *Tinospora cordifolia* stem, biogenic ZnO-NPs, and ZnO-NPs, as determined by MTT assays. The data represent the mean values ± SD of three independent experiments performed in triplicate. Statistical values were calculated using ANOVA: single factor. *p ≤ 0.05, **p ≤ 0.01, ***p ≤ 0.001, ns P > 0.05. The results were presented as the mean standard deviation of three independent experiments. TM, methanol extract of *T. cordifolia* stem; ZnO-NPs TM, biogenic ZnO-NPs conjugated with methanol extract of *T. cordifolia* stem.

The cytotoxic activity of biogenic ZnO-NPs was performed per the MTT assay protocol. As shown in (Figure 8), HCT-116 and Caco-2 as well as a control cell line HEK-293 were treated with different concentrations of biogenic ZnO-NPs (5, 25, 75, 150 and 300 μg/ml) for 24 hours. The biogenic ZnO-NPs showed high efficacy compared with the corresponding crude 90% methanol extract of the stem of *T. cordifolia*. The IC_50_ of biogenic ZnO-NPs for HCT-116 and Caco-2 cell lines were 31.419 ± 0.682 μg/ml and 36.675 ± 0.916 μg/ml, and the control cell line (HEK-293) was 231.867 ± 40.200 μg/ml, respectively. The result showed that the IC_50_ for the crude 90% methanol extract of *T. cordifolia* stem is significantly higher than its corresponding biogenic ZnO-NPs. The IC_50_ for HCT-116 and Caco-2 cell lines was 260.151 ± 1.257 μg/ml and 286.399 ± 12.387 μg/ml, and the control cell line (HEK-293) was 303.860 ± 8.473 μg/ml, respectively. The IC50 of positive control doxorubicin for HCT-116 and Caco-2 cell lines was 3.132 ± 0.35 μg/ml and 3.304 ± 0.523 μg/ml.

### 3.8. Biogenic ZnO-NPs' apoptotic effect

#### 3.8.1. Double staining with AO/EtBr

**Figure 9:**
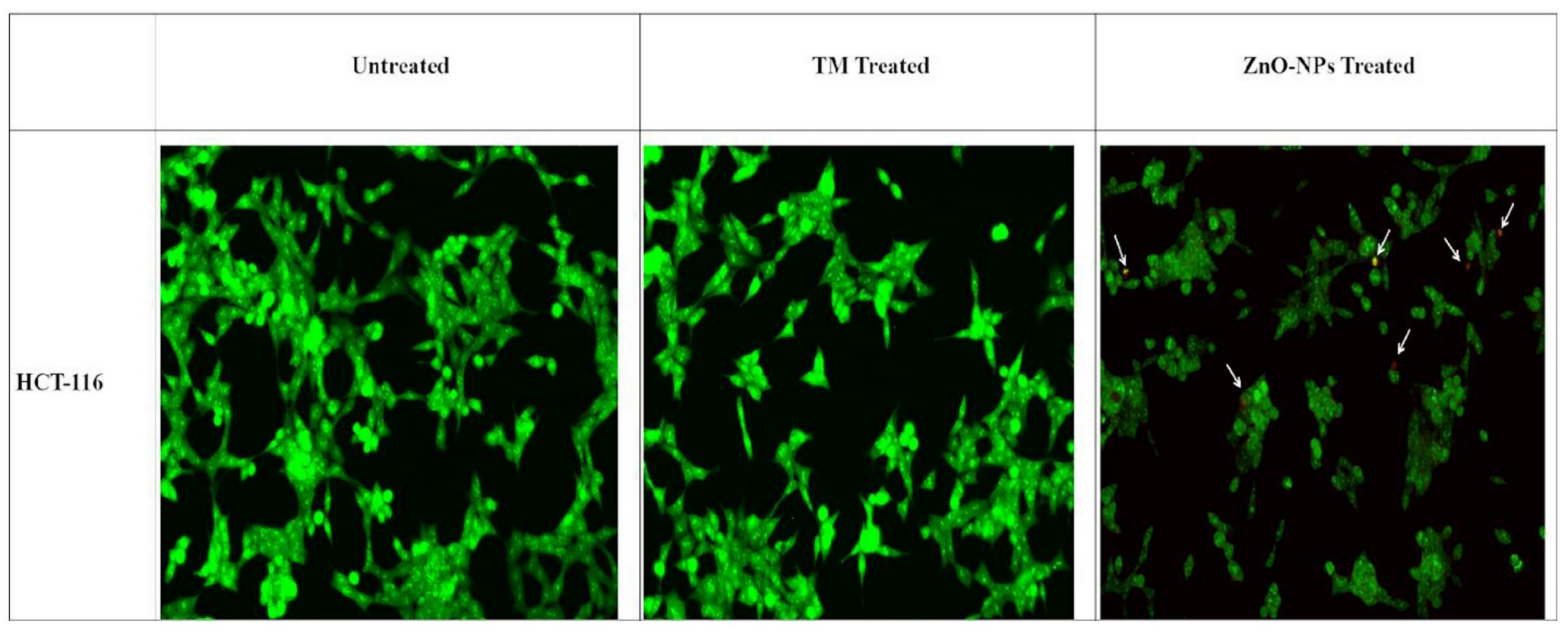
AO/EtBr double staining of HCT-116 cell lines. Live cells are uniformly green, whereas apoptotic cells are stained orange-red due to chromatin condensation and loss of membrane integrity. Pictures were taken at magnification of ×40. TM, methanol extract of *T. cordifolia* stem; ZnO-NPs TM, biogenic ZnO-NPs conjugated with methanol extract of *T. cordifolia* stem.

AO/EtBr double staining was used to check the morphological changes in HCT-116 cell lines 24 hours post-treatment with biogenic ZnO-NPs. As shown in (Figure 9), the untreated cells showed no significant change. Biogenic ZnO-NPs treated HCT-116 cell lines showed substantial red-pale color due to apoptotic cell death, resulting in highly condensed nuclear content. Further cells in the early apoptotic stage showed a change in shape and bubbling.

#### 3.8.2. Flow Cytometry Analysis of the Annexin/PI Assay

**Figure 10:**
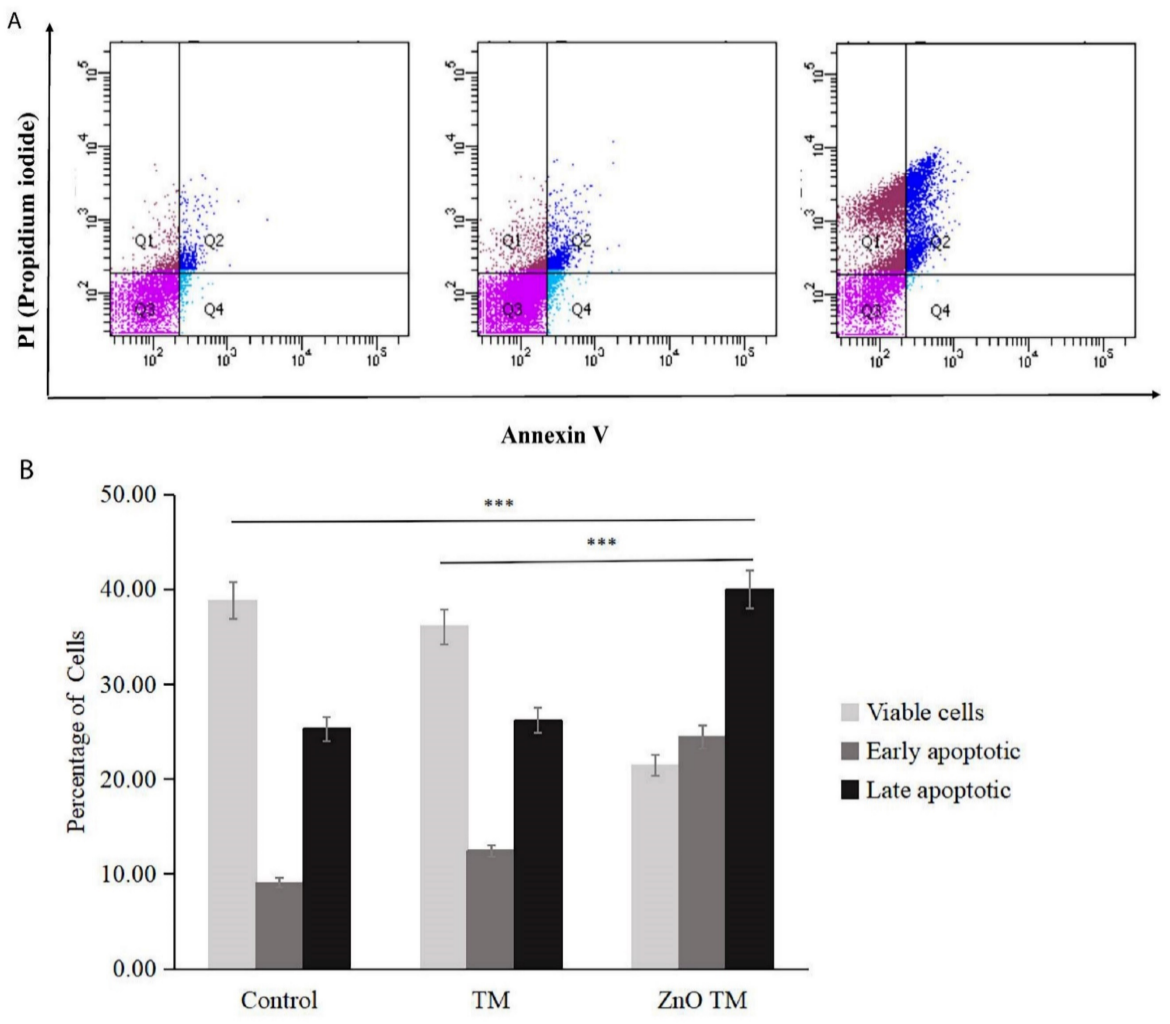
Flow cytometric analysis of Apoptosis. (A) Apoptosis analysis by Annexin-V/PI double staining of untreated HCT-116 cells, HCT-116 treated with biogenic ZnO-NPs and crude extract TM for 24 h. The bottom right quadrant represents Annexin V-FITC-stained cells (early-phase apoptotic cells) and the top right quadrant represents PI-and Annexin V-FITC-dual-stained cells (late-phase apoptotic/necrotic cells). (B) Bar graph representing percentages of viable cells (Annexin-V negative, PI negative), early-apoptotic cells (Annexin-V positive, PI negative) and late-apoptotic dead cells (Annexin-V positive, PI positive) as measured by flow cytometry. TM, methanol extract of *T. cordifolia* stem; ZnOTM, biogenic ZnO-NPs conjugated with methanol extract of *T. cordifolia* stem. Data are shown as means ± s.d. At least three independent experiments were performed., *p < 0.05.

Annexin V/ Propidium iodide assay analysis was carried out using flow cytometry to investigate the biogenic ZnO-NP's ability to induce apoptosis. After 24 hours of post-treatment incubation at 37°C and 5% CO_2_, HCT-116 cell lines were stained and analyzed per the manufacturer's protocol. As shown in (Figure 10), either early or late apoptosis was activated.

Flow cytometry analysis results of HCT-116 treated with IC_50_ concentration for both biogenic ZnO-NPs and crude plant extract, as well as non-treated cells in a 6-well plate. According to the flow cytometry results, the average cell percentage of live, apoptotic and necrotic cells after treatment were analysed. ZnO-NPs treated cells showed that early apoptosis was 0.97%, late apoptosis was 28.43 %, and necrosis was 33.47 respectively. The early apoptotic, late apoptosis, and necrotic cell percentages for crude plant-only treated cell lines were 5.20%, 8.47% and 4.07%, respectively. Whereas for untreated HCT-11 cell lines were 4.30%, 9.1% and 6.80%, respectively. There was a significant difference between the control and biogenic ZnO-NPs treated cells (<0.005). The findings showed that biogenic ZnO-NPs induce apoptotic death in HCT-116 cell lines.

#### 3.8.3. Gene expression analysis/ qRT-PCR

**Figure 11:**
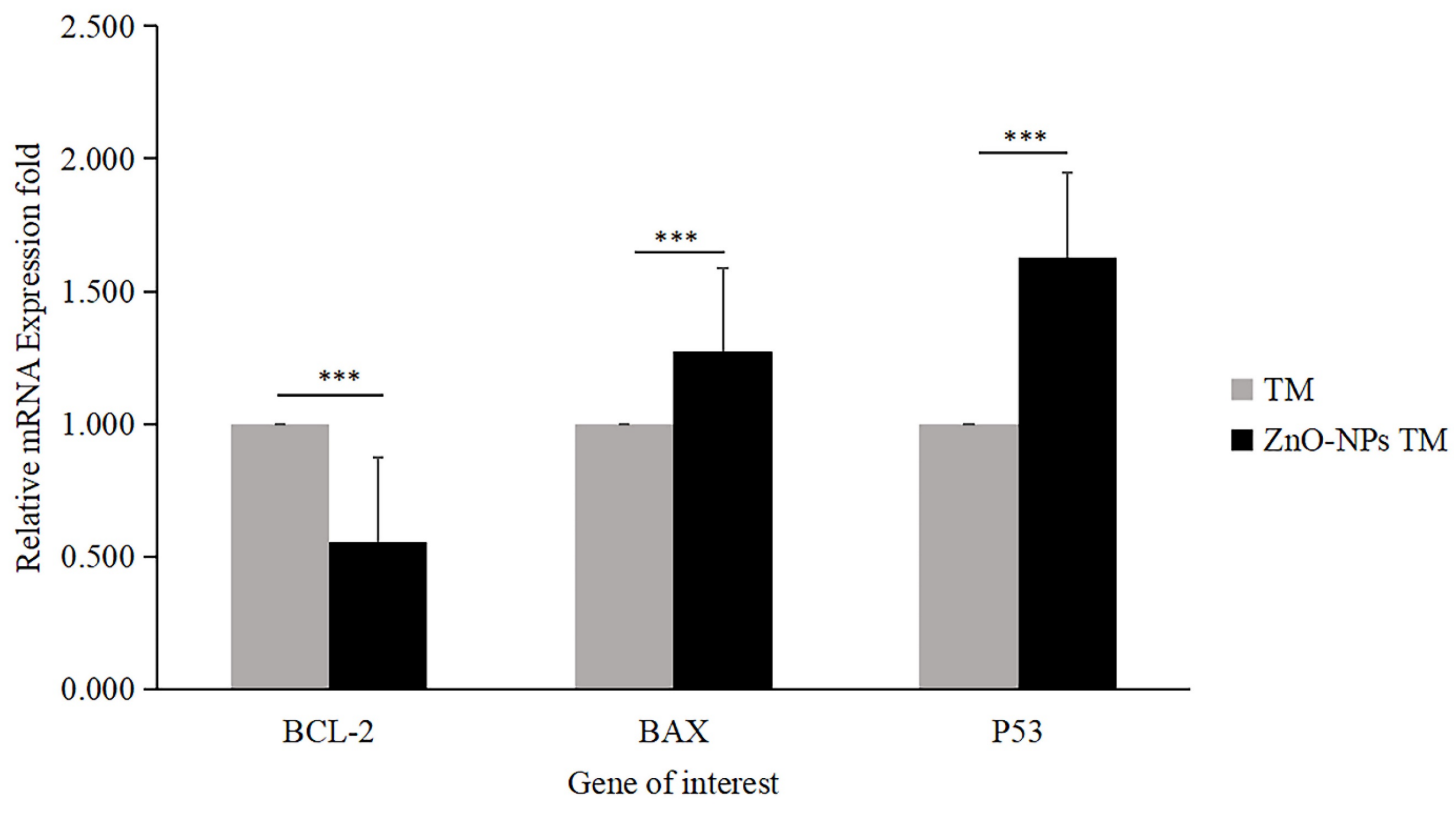
Relative mRNA fold change determined by quantitative real-time PCR (qRT-PCR) analysis of apoptotic markers (BCL-2, BAX, and P53). Data were normalized with GAPDH expression. HCT-116 cell lines were treated with methanol extract of the stem of *T. cordifolia* (TM) and biogenic ZnO-NPs (ZnO-NPs TM) for 24 hours. The results were analyzed by One-way Analysis of Variance (ANOVA) and Tukey's post-hoc-test to determine a significant difference between the treatments (P < 0.05). *p ≤ 0.05, **p ≤ 0.01, ***p ≤ 0.001, ns P > 0.05 were used to represent all data.

The expression level of proapoptotic genes and antiapoptotic gene mRNA levels determines apoptosis. Bax is essential for cytochrome C release and subsequent caspase activation 38. Hence, to determine the apoptotic efficacy of biogenic ZnO-NPs, cells were collected using triazole after 24 hours of exposure to biogenic ZnO-NPs and crude plant extract at an IC_50_ concentration. To determine the change in the mRNA level of the proapoptotic (Bax) and antiapoptotic (Bcl-2) markers, quantitative RT-PCR was used. As shown in Figure 11, the fold change in mRNA level for Bax and P53 was 1.273 and 1.629, respectively, while Bcl-2 was 0.556fold for biogenic ZnO-NPs compared to crude plant extract treated cells. The result revealed that expression of Bax and P53 was significantly enhanced (p <0.001) 24 hours post-treatment of HCT-116 cell lines.

### 3.9. Measurement of ROS generation

HCT-116 cells treated with biogenic ZnO-NPs displayed elevated ROS formation compared to cells treated with crude plant extract and non-treated controls. The result is a fold increase over non-treated HCT-116 cell lines. In comparison to control cells, biogenic ZnO-NPs treated cells increased ROS production by a factor of 2.261. Contrarily, cells treated with crude extract exhibited just a 1.283-fold increase in ROS formation.

### 3.10. Effect of ZnO-NPs on Mitochondrial Membrane Potential

**Figure 13:**
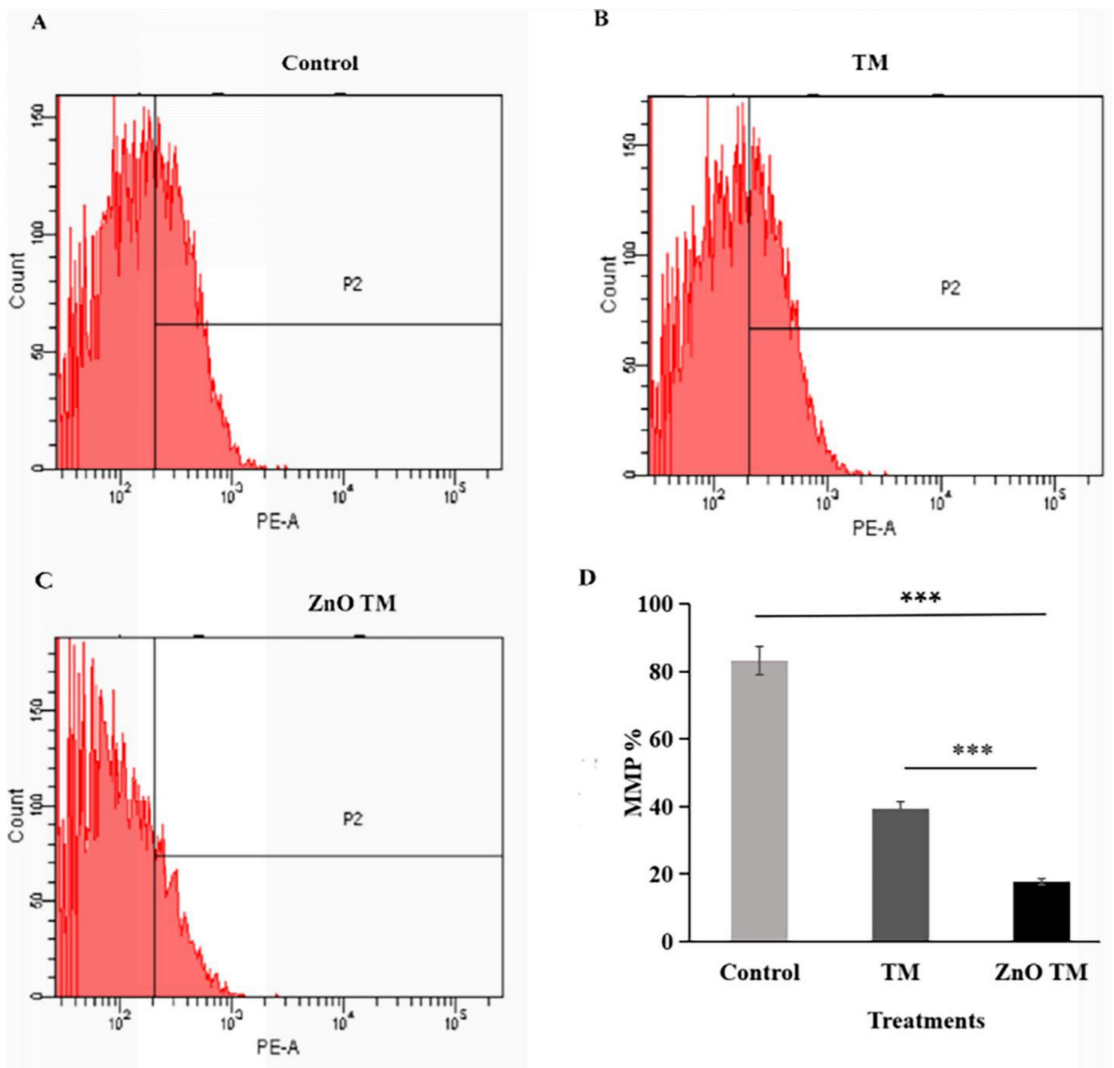
Mitochondrial membrane potential analysis of HCT-116 cell lines. The percentage of MMP of cells were assessed by flowcytometry. The mitochondrial membrane potential (MMP) of untreated HCT-116 cells, HCT-116 treated with biogenic crude extract TM and ZnO-NPs after 24 h of treatment. (A) Untreated cells (B) HCT-116 cell lines were treated with crude methanol extract of stem of *T.cordifolia* and (C) Biogenic ZnO-NPs treated cells for 24 hours. (D) Graph of MMP % of treated cell lines in relation to control. The X-axis (FL-1channel) of flow cytometry results indicated the green fluorescence intensity (JC-1 monomers) and the Y-axis (FL-2channel) was used to detect the red fluorescence (JC-1aggregates). The P2 (region 2) encloses the low MMP cell population. The mitochondrial depolarization is indicated by a decrease in the red/green fluorescence intensity ratio. The ratio of the red/green fluorescence intensity was recorded to determine the MMP level of each sample. Data are shown as means ± s.d. At least three independent experiments were performed., *p < 0.05.

The result showed that HCT-116 cells treated with biogenic ZnO-NPs showed a significant decrease in JC-1 staining (17.63 %), which was confirmed to reflect the loss of the mitochondrial membrane potential, in comparison with cells treated with crude plant extracts and untreated/ control cells (39.4% and 83.2% respectively). Untreated cells form aggregate and emits red fluorescence, indicating increased mitochondrial membrane potential, whereas cells treated with biogenic ZnO-NPs exhibited low green fluorescence, as shown in (Figure 13). This suggests that biogenic ZnO-NPs induce cell death through the mitochondrial-mediated mechanism.

### 3.11. Biogenic ZnO-NPs induced caspase-dependent apoptosis

**Figure 14:**
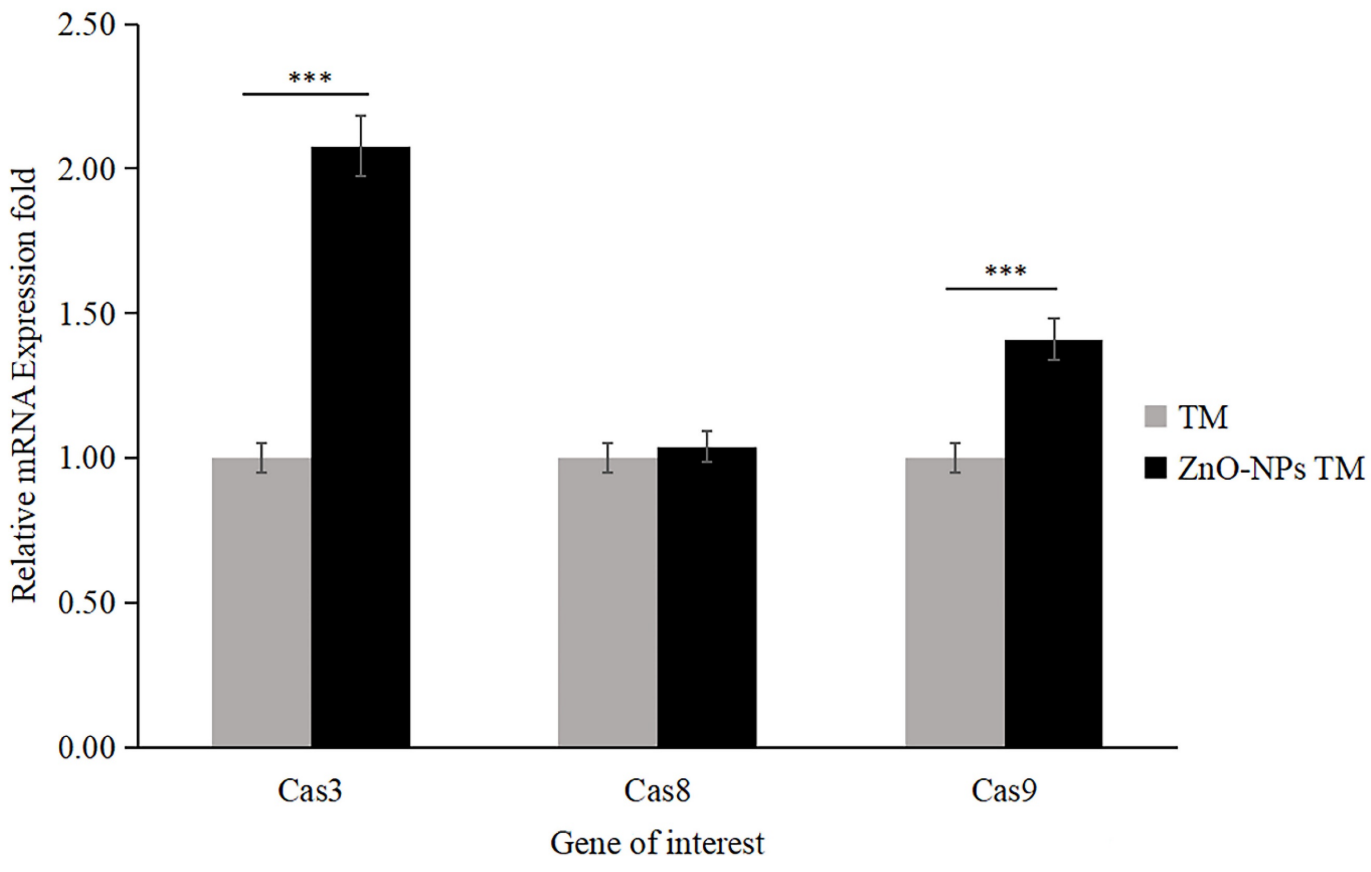
qRT-PCR analysis of caspase activities (Caspase-3, Caspase-8, and Caspase-9; data were normalized with GAPDH expression) determined relative mRNA fold change. HCT-116 cell lines were treated with methanol extract of the stem of *T. cordifolia* (TM) and biogenic ZnO-NPs (ZnO-NPs TM) for 24 hours. The results were analyzed by One-way Analysis of Variance (ANOVA) and Tukey's post-hoc-test to determine a significant difference between the treatments (P < 0.05). All data were represented mean ± SD, *p ≤ 0.05, **p ≤ 0.01, ***p ≤ 0.001, ns P > 0.05.

The apoptosis analysis determined by RT-qPCR showed that the expression level of mRNA of caspase-3 and caspase-9 significantly increased by 2.078 and 1.41 folds, respectively, in biogenic ZnO-NPs treated HCT-116 cell lines compared to only crude extract treated cells (Figure 14). Meanwhile, the expression level of mRNA of caspase-8 was 1.039, which does not show any significant change. The data were presented as the mean ± SEM.

### 3.12. Biogenic ZnO-NPs inhibit tumor growth *in vivo.*

**Figure 15:**
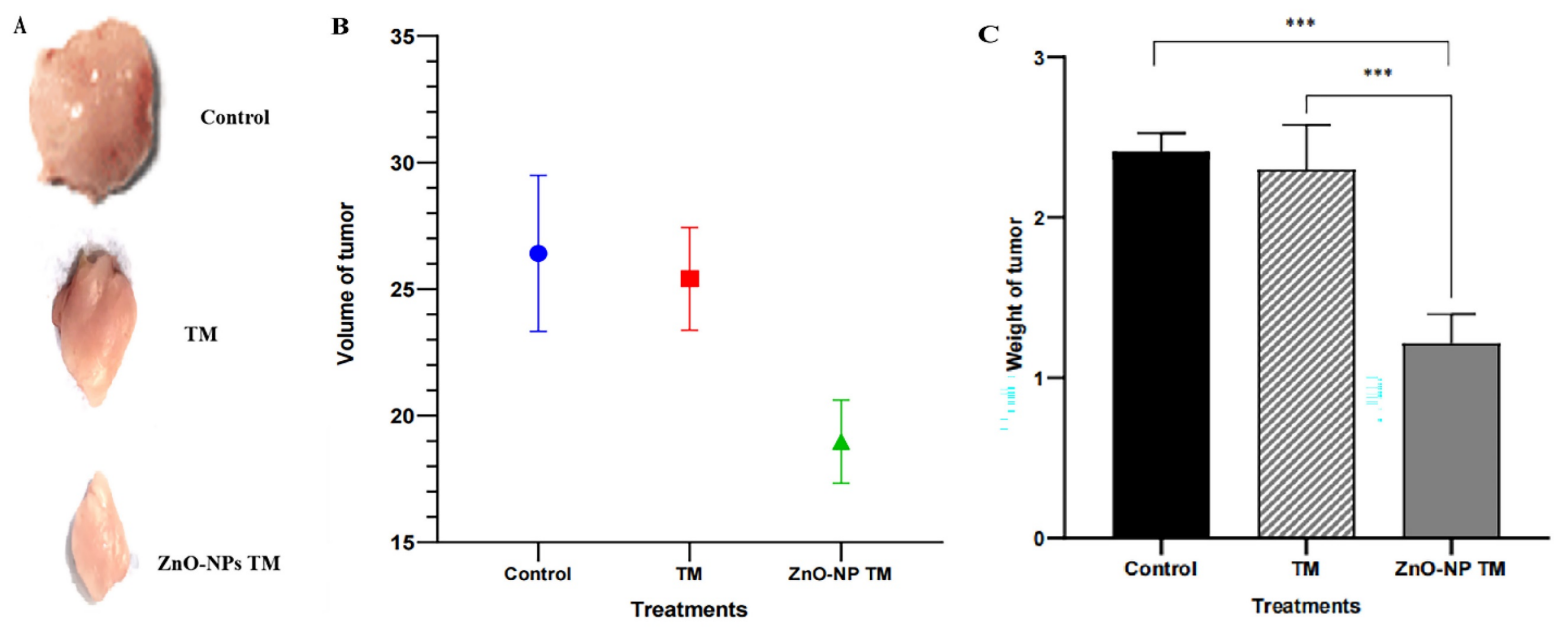
*In vivo* anti-tumor efficacy of biogenic ZnO-NPs and its corresponding crude plant extracts. (A) The photo of excised tumors at the end of the test. (B) Tumor weight at the end point of the treatment. (C) The volume of the harvested tumor (For interpretation TM= *T. cordifolia* stem methanol extracts, ZnO-NPs TM= biogenic ZnO-NPs synthesized from *T. cordifolia* stem methanol extracts. (*p < 0.05 and **p < 0.01, and ***p < 0.001).

The tumor growth slows down on treating biogenic ZnO-NPs compared to other groups. After two weeks of treatment, the excised tumor size and weight were reduced in the biogenic ZnO-NPs treated mice group. Figure 15 shows a significant reduction in tumor weight and volume in mice treated with biogenic ZnO-NPs compared to their corresponding crude plant extracts (p < 0.001). The Mice group treated with biogenic ZnO-NPs synthesized from the extract of *T. cordifolia* stem decreased by 1.499-fold in volume and 1.528 in weight compared to the crude extract of *T. cordifolia* stem treated group. These data suggest an anti-tumor growth effect of biogenic ZnO-NPs synthesized from *T. cordifolia* methanol extract stem *in vivo* studies.

## 4. Discussion

Methanol (90%) extract of *Tinospora cordifolia* stem was used to synthesize ZnO-NPs successfully. We demonstrate the promising potential of this traditional medicine for the safer, more economical, and more eco-friendly synthesis of ZnO-NPs. Plants were thought to be an unrivalled source of a new drug in the future, and this value was linked to their active biocomponents 32. The green synthesis of NPs depends on plants' ability to store metal ions and use active phytoconstituents as bio-reductants and stabilizers 33. Several spectroscopy and microscopy techniques, such as UV-Vis, FTIR, XRD, SEM, and TEM, were used to study and describe the biogenic ZnO-NPs made from the methanol extract of the *T. cordifolia* stem. These spectroscopy and microscopy techniques offer valuable insights into materials' properties, composition, and structure 34. UV-vis spectroscopy is a widely used tool for assessing the optical properties of synthesized NPs. So, the strong absorption spectrum of biogenic ZnO-NPs at 374 nm suggests the presence of ZnO-NPs with distinct peaks. This shows that the biomolecules in the plant extracts were used to reduce and stabilize the synthesis of pure biogenic ZnO-NPs. A similar result was also reported by Selim et al.,2020 35; green ZnO-NPs synthesized using an aqueous Deverra tortuosa extract displayed a distinctive absorption spectra peak at 374 nm. Previous studies have indicated that ZnO-NPs display an absorption peak between 330 and 460 nm 36, 37.

The presence of phytochemicals in the extract, as well as the purity and makeup of the synthesized nanoparticles, were both determined using FTIR. The ZnO-NPs surface can interact with the plant's phytochemicals, such as alcohols, phenols, amines, carboxylic acids, and others that help to stabilize the nanoparticles. The peaks signify the functional groups that are conjugated with ZnO-NPs. The spectra of synthesized biogenic ZnO-NPs correspond with previous findings 38, 21.

The XRD diffraction pattern of the biogenic ZnO-NPs demonstrated the purity of ZnO-nanocrystalline synthesis. The NPs appeared well crystallized and hexagonal in a structure that agrees with the worldwide standard JCPDS No. 00-036-1451 24. The average crystallite size of ZnO-NPs was similar to results reported by Tiwari et al., 39; the average crystalline size of ZnO-NPs synthesized chemically and biologically were 47 nm and 55 nm, respectively. Furthermore, Faisal et al., 40 reported that the green synthesized ZnO-NPs from aqueous extracts of *Myristica fragrans* fruit had an average crystallite size of 41.23 nm.

Microscopy analysis of biogenic ZnO-NPs was conducted to determine the surface morphology of the biogenic ZnO-NPs. The shape of NPs plays a crucial role in their efficacy 41. Both the analysis (SEM and TEM) demonstrated that ZnO-NPs were synthesized. SEM analysis revealed that the particles have a sphere-shaped morphology with a high degree of aggregation and a spongy-like appearance topographically. The particle size varies, as shown in figure 6. Other researchers reported similar results 41. The TEM analysis showed that ZnO-NPs were synthesized at different magnifications by demonstrating the hexagonal plane.

Furthermore, the prominent bright rings are visible in the selected area of electron diffraction, as shown in Figure 7. These findings align with previous results 42. The analysis showed that the average particle size is very small compared to XRD's average crystalline size. The parameters we used to figure out how biogenic ZnO-NPs are made show that the structure and shape of the synthesized nanoparticles match what has been found before 43.

Previous findings showed that green-synthesized ZnO-NPs are effective in treating colorectal cancer. Due to their capping of phytochemicals, plant-mediated ZnO-NPs have shown promising anticancer efficacy, such as colon, cervical, breast, and lung cancer 44, 45. This study examined how well biogenic ZnO-NPs made from a methanol extract of T. cordifolia stems could treat colorectal cancer cell lines. The cancer cell cytotoxicity test revealed that biogenic ZnO-NPs had a high cell inhibition efficacy for HCT-116 and Caco-2 cell lines, which is consistent with previous results reported 35, 46. In line with our findings, Berehu et al., 23 reported that ZnO-NPs synthesized from methanolic and ethanolic extracts of *Swertia chirayita* leaves showed cytotoxic activities against colorectal cancer cell lines.

A study claimed by Selim et al., 35 investigated the anticancer efficacy of green synthesized ZnO-NPs from *D. tortusa* against Caco-2 and A549 cell lines. The cytotoxic effects of the crude plant extract and synthesized ZnO-NPs were much stronger on the cancer cell lines Caco-2 and A549 than on the normal cell line WI38. But the IC_50_ values of synthesized ZnO-NPs for A549 and Caco-2 cell lines are 83.47 g/mL and 50.81 g/mL. Similarly, Dulta et al., 47 reported that ZnO-NPs synthesized by rhizome extract of *Bergenia ciliata* showed an IC_50_ value of 124.3 μg/mL against HT-29 cell lines. These results indicated that biogenic ZnO-NPs synthesized from plant sources have cytotoxic activity against cancer cell lines.

Apoptosis is directly linked to the regulation of the cell cycle. If the apoptotic mechanism is absent, uncontrolled cell proliferation will be there, which promotes cancer progression. Thus, in this study, we conducted an Annexin V/propidium iodide assay, AO/EtBr staining, and evaluated the mRNA levels of Bax, Bcl-2, and P53 to determine the ability of synthesized biogenic ZnO-NPs to induce apoptosis in HCT-116 cell lines. The initial stages of apoptosis include shrinkage of cell detachment and bubbling of cells deemed to undergo apoptosis 23. AO/EtBr double staining was used to check the morphological changes in HCT-116 cell lines. Biogenic ZnO-NP-treated HCT-116 cell lines showed significant red-pale color due to apoptotic cell death, resulting in highly condensed nuclear content. Further cells in the early apoptotic stage showed a change in shape and bubbling. Several findings showed the same result when cells undergo apoptosis: the cells' DNA becomes thick, fragmented, and condensed into chromatin 48, 49.

In a related study, annexin V-FITC/PI FACS analysis showed that ZnO-NPs-treated cells showed relatively high apoptosis percentage. The apoptotic, cell percentages for crude plant-only treated cell lines were low. In our annexin V-FITC/PI FACS analysis study, the percentage of HCT-116 cell lines entering early apoptosis was higher in ZnO-NPs-treated cells compared to the control and crude extract-treated cells. However, the percentages of necrotic cells were elevated in control and crude extract-treated cells compared to ZnO-NPs-treated HCT-116 cell lines. The cytoplasmic and lysosomal membranes are maintained during apoptosis 50. On the other hand, necrosis results in unregulated inflammatory cellular responses characterized by cell swelling and membrane permeabilization. Apoptosis during cancer treatment has drawn much attention because it is thought to be a regulated and controlled process.

Furthermore, we quantified the mRNA level using qRT-PCR for Bax and Bcl-2 mRNA levels. As shown in Figure 11, the fold change in mRNA level for Bax and P53 were high, while Bcl-2 was reduced for biogenic ZnO-NPs compared to crude plant extract-treated cells. Our findings indicated that the expression of Bax and P53 was significantly elevated 24 hours post-treatment of HCT-116 cell lines. Other studies that saw results similar to these indicated a significant rise in the levels of Bax in Osteosarcoma cell lines (MG-63) treated with synthesized ZnO-NPs from *Rehmanniae Radix* 24. Similar findings showed that laryngeal cancer cells (Hep-2) treated with synthesized ZnO-NPs from *Marsdenia Tenacissima* expressed a higher Bax and a lower Bcl-2 level of mRNA 51, 52.

Numerous studies have demonstrated that ROS generation causes ZnO-NPs to be cytotoxic. During apoptosis, MMP is lost, which depolarizes the membrane. An increase in ROS generation causes a higher incidence of apoptosis in cancer cells 53, 54. Our study showed that cells treated with biogenic ZnO-NPs generated ROS more than untreated cells. In contrast, ROS production was increased by just 1.283-fold in cells treated with the crude extract. Different authors reported similar results 25. Biogenic ZnO-NPs treated cells trigger ROS generation consecutively, leading to impairment of MMP. Previous research found that altered ROS homeostasis harmed proteins, DNA, and lipids. As a result, any interventions that promote ROS generation may result in greater mitochondrial damage and an increased apoptosis rate 55. In line with this, our findings showed that HCT-116 cells treated with biogenic ZnO-NPs showed a significant decline in JC-1 staining, which was confirmed to reflect the loss of the MMP, in comparison with cells treated with crude plant extracts and untreated/ control cells. The result corresponds with the findings of research conducted by Cheng et al., 24 suggested that ZnO-NPs from *Rehmanniae Radix* (RR) induce apoptosis on MG-63 cells through stimulation of ROS generation., which disrupts the MMP 56.

According to different studies, targeting the apoptotic pathways is the most important therapeutic approach for cancer treatment. Apoptosis happens through several mechanisms, including an alteration in the internal mitochondrial membrane that activates the caspase cascade. There are two main apoptotic pathways, intrinsic and extrinsic. Each pathway triggers caspase initiators (caspase-8 and caspase-9), activating caspase-3, the executioner 57. Caspase-8 activates when the extrinsic pathway is induced, resulting in the activation and cleavage of caspase-3. The intrinsic pathway, in contrast, prerequisites the mitochondrial membrane alteration and cytochrome c release. The activation of Bax and Bcl-2 serve as triggers for cytochrome C release, consecutively activating caspases. Apoptosis is initiated when cytochrome c is released and interacts with the Apaf-1 protein, which activates caspase-9 and promotes the activation and cleavage caspase-3. These results indicated the potent activation of caspase-3 and caspase-9 by biogenic ZnO-NPs. Wang et al., 52 published data showing that ZnO-NPs synthesized from *M. tenacissima* promote the over-expression of Bax, caspase-3, and caspase-9 and the down-regulation of Bcl-2 in Hep-2 cell lines. Similar studies were reported in different articles 58.

An *in vivo* study indicated visible anti-tumor activity of biogenic ZnO-NPs compared to their crude plant extract in BALB/c mice. The growth of tumor slows down on the treatment of biogenic ZnO-NPs compared to other groups. After two weeks of treatment, the excised tumor size and weight were reduced in biogenic ZnO-NPs treated mice. There was a significant reduction in tumor weight and volume in mice treated with biogenic ZnO-NPs compared to their corresponding crude plant extracts. (p < 0.001). Mice group treated with biogenic ZnO-NPs synthesized from the extract of *T. cordifolia* stem tumor volume and weight decreased by 1.499 and 1.528 folds, respectively, compared to only crude extract of *T. cordifolia* stem treated groups. According to the findings, biogenic ZnO-NPs treatment was able to limit tumor growth significantly. The *in vivo* findings supported the *in vitro* findings that biogenic ZnO-NPs exhibit preferential anticancer activity.

Our findings showed that biogenic ZnO-NPs mediated apoptosis involves the intrinsic apoptotic pathway. The caspase-dependent route was the mechanism by which biogenic ZnO-NPs caused cell death. Overall, we suggest that biogenic ZnO-NPs mediated caspase-dependent apoptosis in HCT-116 cell lines is related to ROS-induced mitochondrial membrane potential disruption.

## 5. Conclusion

Our findings show that biogenic ZnO-NPs synthesized from methanol extract of *Tinospora cordifolia* stem successfully generated ROS, altered MMP, and qRT-PCR analysis confirmed the mRNA level of Bax (proapoptotic gene) increased on treated HCT-116 cell lines. In contrast, the mRNA level of Bcl-2 (antiapoptotic gene) decreased. This implies that cellular apoptosis was significantly induced. The caspase-dependent route was the mechanism by which biogenic ZnO-NPs caused cell death. Overall, we suggest that biogenic ZnO-NPs mediated caspase-dependent apoptosis in HCT-116 cell lines is related to ROS-induced mitochondrial membrane potential disruption. Considering all of this evidence, green synthesized ZnO-NPs from the methanol extract of *Tinospora cordifolia* stem could be an alternative anticancer therapeutic regime in colorectal cancer.

## Supplementary Material

Supplementary figure and table.

## Figures and Tables

**Figure 1:**
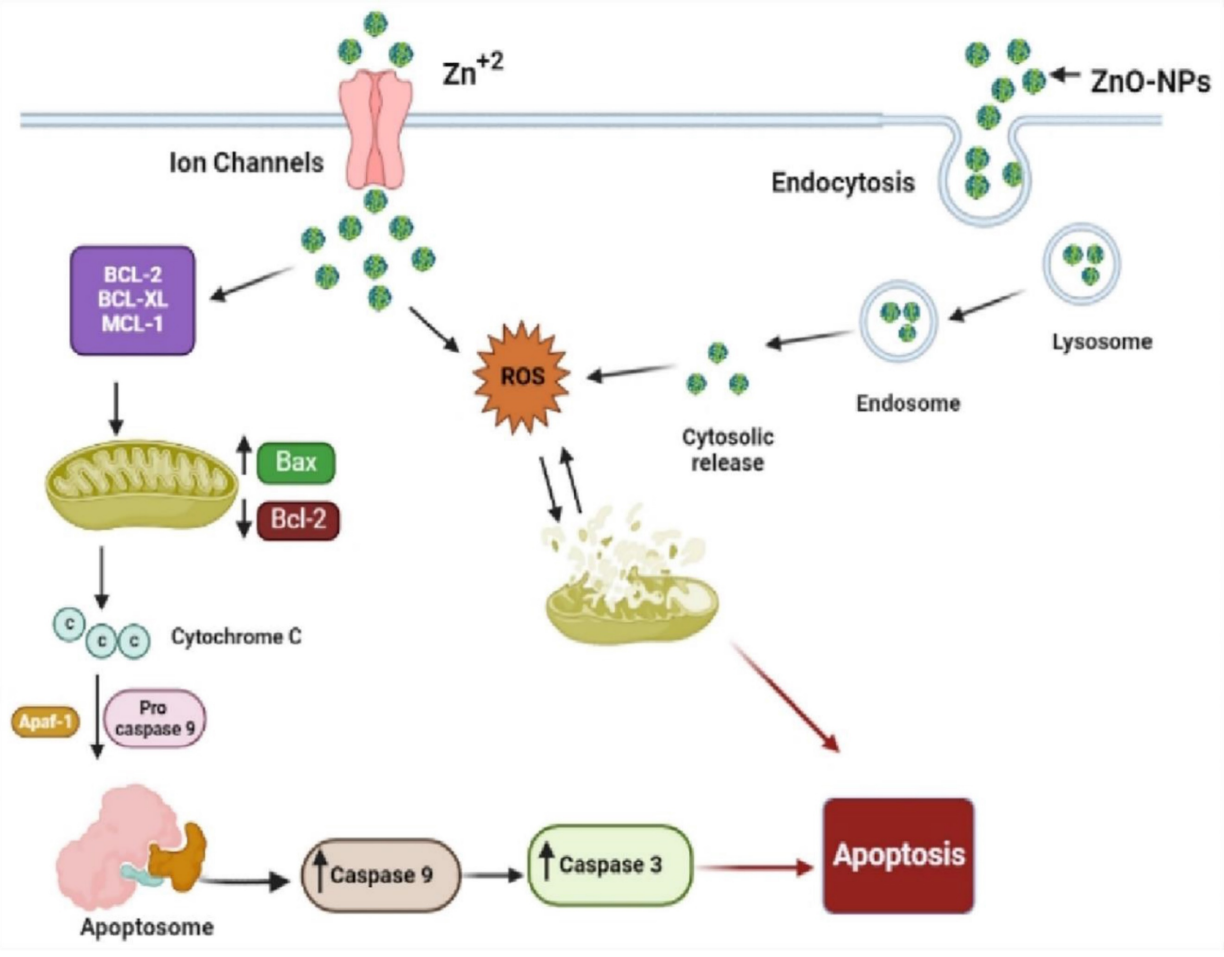
Schematic representation of the possible mechanism of biogenic ZnO-NPs-induced apoptosis.

**Figure 12:**
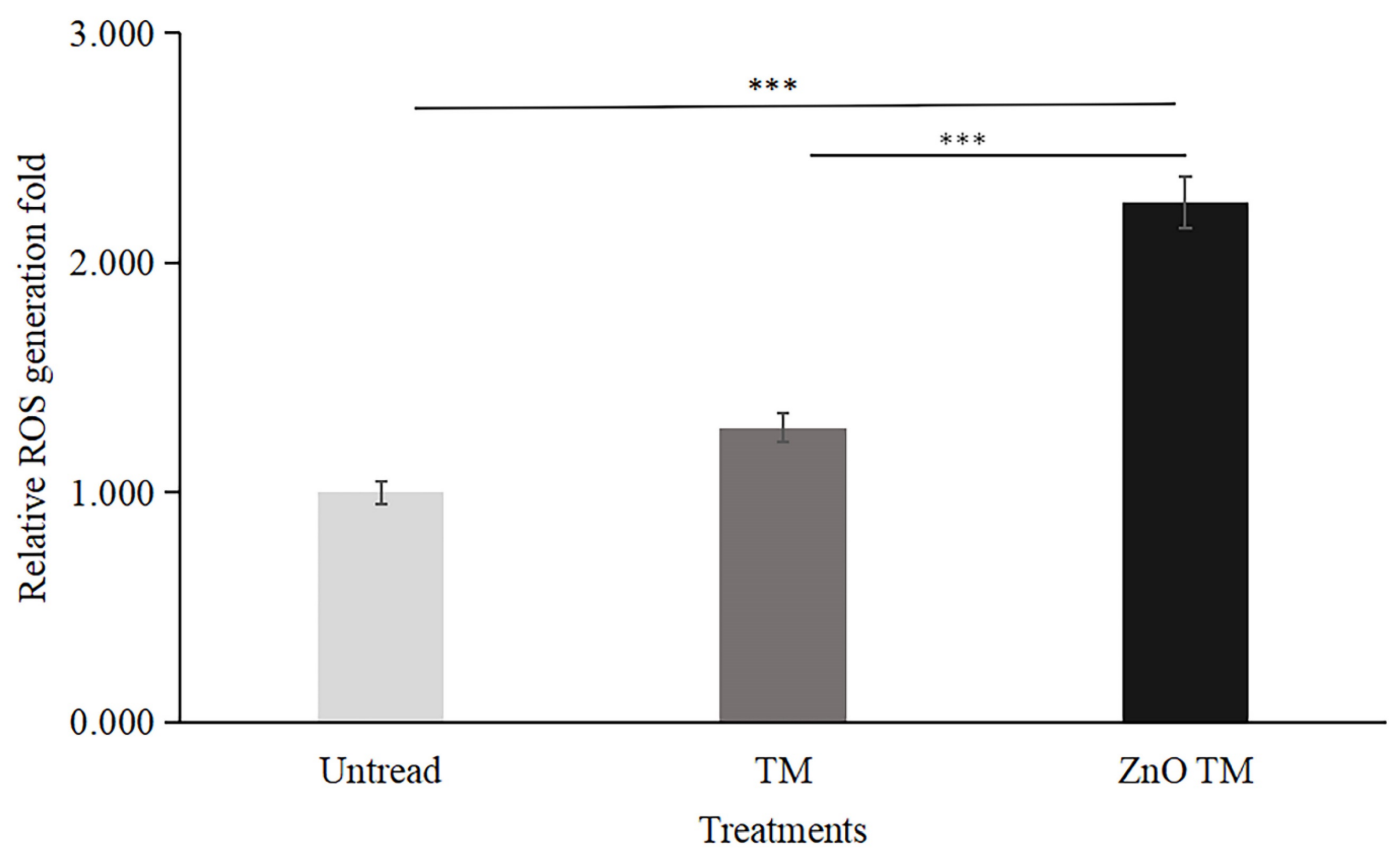
Quantative representation of ROS generation fold. Data are presented as fold increase over control (no treatment). Bars represent the mean ± standard deviation of three independent experiments. *, P < 0.05; **, P < 0.01; and ***, P < 0.001. TM, methanol extract of *T. cordifolia* stem; ZnO-NPs TM, biogenic ZnO-NPs conjugated with methanol extract of *T. cordifolia* stem.
