# Supplementary material for: Biogenic Zinc Oxide Nanoparticles synthesized from Tinospora Cordifolia induce oxidative stress, mitochondrial damage and apoptosis in Colorectal Cancer
Source: Nanotheranostics. 2024 Mar 9;8(3):312–29. doi: 10.7150/ntno.84995 (PMC10988208; doi:10.7150/ntno.84995)
Supplement: Supplementary file 1 — Supplementary figure and table. [file ntnov08p0312s1.pdf]

Biogenic Zinc Oxide Nanoparticles synthesized from *Tinospora Cordifolia* induce oxidative stress, mitochondrial damage and apoptosis in Colorectal Cancer

Hadgu Mendefro Berehu[1], **Srinivas Patnaik**[1]*

Disease Biology Laboratory, School of Biotechnology KIIT Deemed to Be University, Odisha, India

Supplementary Table S1: List of phytochemical compounds detected in the Methanol extract of *Tinospora Cordifolia* using GC-MS.

Peak #	Compound Name	PubChem CID	Molecular formula
1	Diacetyl sulphide	76708	$C_4H_6O_2S$
2	Cyclobutane, methyl-	11725	C_5H_{10}
3	Cyclobutane, 2-ethyl-1-methyl-3-propyl	550796	$C_{10}H_{20}$
4	2,4-Dihydroxy-2,5-dimethyl-3(2H)-furan-3	538757	$C_6H_8O_4$
5	l-Alanine, n-propargyloxycarbonyl-, hexy	7174	$C_{10}H_{13}NO_2$
6	2,3-Dihydro-2,5-dihydoxy-6-methyl-4-H-pyran-4-one	119838	$C_6H_8O_4$
7	1,1,2-Triacetoxyethane	76325	$C_8H_{12}O_6$
8	4-Hydroxy-2-methylacetophenone	70133	$C_9H_{10}O_2$
9	Phenol, 2,6-dimethoxy	7041	$C_8H_{10}O_3$
10	Benzaldehyde, 3-hydroxy-4-methoxy	94857	$C_8H_7NO_5$
11	Phenol, 2-methoxy-4-(1-propenyl)-	853433	$C_{10}H_{12}O_2$
12	Z-8-Methyl-9-tetradecenoic acid	5364410	$C_{15}H_{28}O_2$
13	8-Pentadecanone	13162	$C_{15}H_{30}O$
14	Phenol, 2,6-dimethoxy-4-(2-propenyl)-	226486	$C_{11}H_{14}O_3$
15	4-((1E)-3-Hydroxy-1-propenyl)-2-methoxyphenol	1549095	$C_{10}H_{12}O_3$
16	1,13-Tetradecadien-3-one	337818	$C_{14}H_{24}O$
17	1-Eicosanol	12404	$C_{20}H_{42}O$
18	Phytol, acetate	6428538	$C_{22}H_{42}O_2$
19	8-Pentadecanone	13162	$C_{15}H_{30}O$
20	3,7,11,15-Tetramethyl-2-hexadecen-1-ol	5366244	$C_{20}H_{40}O$
21	n-Hexadecanoic acid	985	$C_{16}H_{32}O_2$
22	1-Eicosanol	12404	$C_{20}H_{42}O$
23	2,2,6,7-Tetramethyl-10-oxatricyclo[4.3.1	536541	$C_{13}H_{22}O_2$
24	9-Octadecenoic acid (Z)-, 2-hydroxy-1-(h	445639	$C_{18}H_{34}O_2$
25	Phytol	5280435	$C_{20}H_{40}O$
26	9,12-Octadecadienoic acid (Z,Z)-	6439696	$C_{36}H_{66}O_4$
27	Cyclopropaneoctanoic acid, 2-[[2-[(2-eth	57346156	$C_{11}H_{20}O_2$
28	Octadecanoic acid, 2-(2-hydroxyethoxy)et	162225	$C_{22}H_{48}N_2O_3$
29	1-Hexacosene	29303	$C_{26}H_{52}$

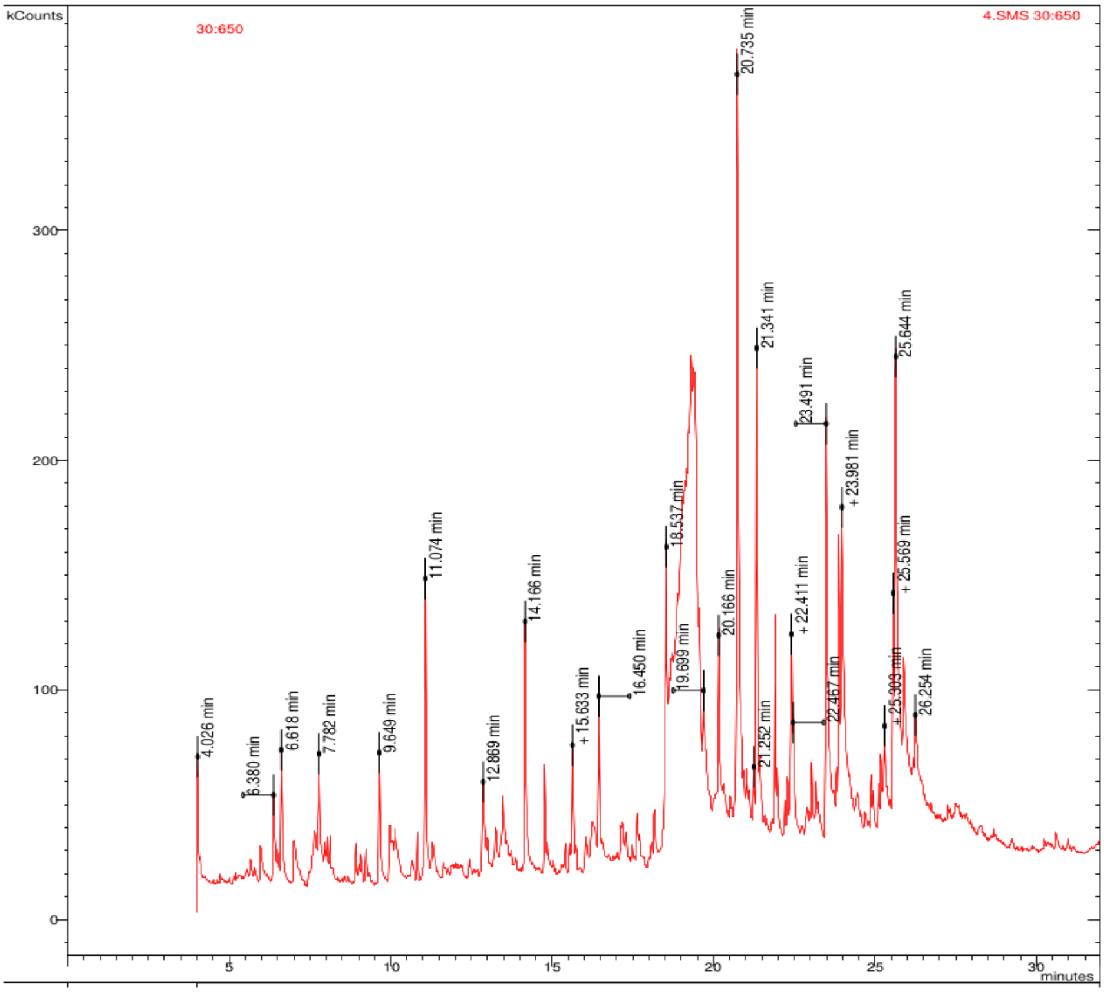

Supplementary Figure S1: GC-MS analysis of phytoconstituents detected in the methanol extract of stem of *Tinospora cordifolia.*
